# Psychosocial and socioeconomic determinants of cardiovascular mortality in Eastern Europe: A multicentre prospective cohort study

**DOI:** 10.1371/journal.pmed.1002459

**Published:** 2017-12-06

**Authors:** Taavi Tillmann, Hynek Pikhart, Anne Peasey, Ruzena Kubinova, Andrzej Pajak, Abdonas Tamosiunas, Sofia Malyutina, Andrew Steptoe, Mika Kivimäki, Michael Marmot, Martin Bobak

**Affiliations:** 1 Department of Epidemiology & Public Health, University College London, London, United Kingdom; 2 Centre for Environmental Health Monitoring, National Institute of Public Health, Prague, Czech Republic; 3 Chair of Epidemiology and Population Studies, Institute of Public Health, Faculty of Health Sciences, Jagiellonian University, Krakow, Poland; 4 Institute of Cardiology, Lithuanian University of Health Sciences, Kaunas, Lithuania; 5 Research Institute of Internal and Preventive Medicine, Branch of the Institute of Cytology and Genetics, SB RAS, Novosibirsk, Russia; 6 Novosibirsk State Medical University, Novosibirsk, Russia; University of Oxford, UNITED KINGDOM

## Abstract

**Background:**

Eastern European countries have some of the highest rates of cardiovascular disease (CVD) mortality, much of which cannot be adequately accounted for by conventional CVD risk factors. Psychosocial and socioeconomic factors may affect risk of CVD, but relatively few studies on this issue have been undertaken in Eastern Europe. We investigated whether various psychosocial factors are associated with CVD mortality independently from each other and whether they can help explain differences in CVD mortality between Eastern European populations.

**Methods:**

Participants were from the Health, Alcohol and Psychological factors in Eastern Europe (HAPIEE) cohort study in Russia, Poland and the Czech Republic, including a total of 20,867 men and women aged 43–74 years and free of CVD at baseline examination during 2002–2005. Participants were followed-up for CVD mortality after linkage to national mortality registries for a median of 7.2 years.

**Results:**

During the follow-up, 556 participants died from CVD. After mutual adjustment, six psychosocial and socioeconomic factors were associated with increased risk of CVD death: unemployment, low material amenities, depression, being single, infrequent contacts with friends or relatives. The hazard ratios [HRs] for these six factors ranged between 1.26 [95% confidence interval 1.14–1.40] and 1.81 [95% confidence interval 1.24–2.64], fully adjusted for each other, and conventional cardiovascular risk factors. Population-attributable fractions ranged from 8% [4%–13%] to 22% [11%–31%] for each factor, when measured on average across the three cohorts. However, the prevalence of psychosocial and socioeconomic risk factors and their HRs were similar between the three countries. Altogether, these factors could not explain why participants from Russia had higher CVD mortality when compared to participants from Poland/Czech Republic. Limitations of this study include measurement error that could lead to residual confounding; and the possibilities for reverse causation and/or unmeasured confounding from observational studies to lead to associations that are not causal in nature.

**Conclusions:**

Six psychosocial and socioeconomic factors were associated with cardiovascular mortality, independent of each other. Differences in mortality between cohorts from Russia versus Poland or Check Republic remained unexplained.

## Abstract 2

Using data from the HAPIEE study, a multicentre prospective cohort study, Taavi Tillmann and colleagues examine the pychosocial and socioeconomic determinants of cardiovascular mortality in Russia, Poland, and the Czech Republic.

## Author summary

**Why was this study done?:**

- Countries in Eastern Europe have unusually high rates of cardiovascular disease, but the causes of this remain unknown.
- Second, studies from around the world have found that people with less education, income, and/or wealth develop more heart disease, but it is unclear why this happens.

**What did the researchers do and find?:**

- We examined data from 20,867 healthy middle-aged adults from Russia, Poland, and the Czech Republic, and asked them a range of questions about stress-related factors. After a median of 7.2 years, 556 of them had died of heart disease.
- We confirmed that people with conventional risk factors (e.g., smoking, high blood pressure, diabetes, physical inactivity, and obesity) were more likely to die from heart disease.
- We also saw that people who were unemployed, single, with less wealth, who rarely saw their friends and relatives, and who are more depressed were more likely to die from heart disease. These associations with heart disease were not explained by the conventional risk factors, and they seemed to be independent of one another.
- After considering country-specific differences in conventional and stress-related factors, we were not able to explain why the risk of cardiovascular death is twice as high in Russia when compared to Poland or the Czech Republic.

**What do these findings mean?:**

- We still do not understand why life expectancy remains so low in many areas of Eastern Europe, such as Russia. The HAPIEE study suggests that alcohol, diet, and stress are unlikely to cause these international differences. New hypotheses are now required to investigate this further.
- There may be at least six stress-related risk factors for heart disease. Each of these could be further investigated to see if they improve clinical risk prediction models, if they are causal, and how they work.

## Introduction

Psychosocial and socioeconomic risk factors, such as unemployment, low social support, and depression, are associated with increased risk of cardiovascular disease (CVD) [1, 2]. Meta-analyses suggest that the excess risk associated with some of these factors can be as high as that for hypertension or raised cholesterol [3–5]. According to the 2016 European guidelines for CVD prevention, these factors are potentially a useful target for intervention [6]. However, the evidence to support this suggestion remains limited.

One weakness in the current evidence base is that most data comes primarily from Western Europe and Northern America, making it unclear to what extent these findings are generalisable elsewhere. For example, Eastern European countries have some of the highest rates of CVD mortality in the world, most of which cannot be adequately accounted for by conventional CVD risk factors [7]. The high rates of CVD mortality in Eastern European countries has a large impact on life expectancy in these countries, which in 2015 was only 65 for men in Russia, compared to 67 for men in India or Cambodia [8]. The 2015 Global Burden of Disease study highlighted how the Eastern European region deviates strikingly from the otherwise tight correlation between life expectancy and socioeconomic development (see figure 10 from reference[9]). This deviation remains an important global health question that could inform development goals elsewhere.

It has been hypothesised that the transition from communism to capitalism (which began in 1989) in Eastern European countries could have exacerbated the influence of psychosocial hazards on CVD [10]. During the early 1990s, countries like Russia experienced a recession many times larger than the recent 2008 global recession. Together with vanishing social welfare, rising crime, political uncertainty, and changing cultural expectations, this period of massive social change coincided, in some countries, with large increases in suicide and CVD mortality, especially among single middle-aged men of low education [11, 12]. However, there have been no prospective studies investigating the role of other psychosocial and socioeconomic risk factors in predicting CVD in this region, such as unemployment, depression, social support, perceived control, material deprivation, material amenities, and loss of social status. Furthermore, literature from other countries is relatively sparse about the extent to which depression and social support might explain socioeconomic differences in CVD mortality.

**Fig 1:**
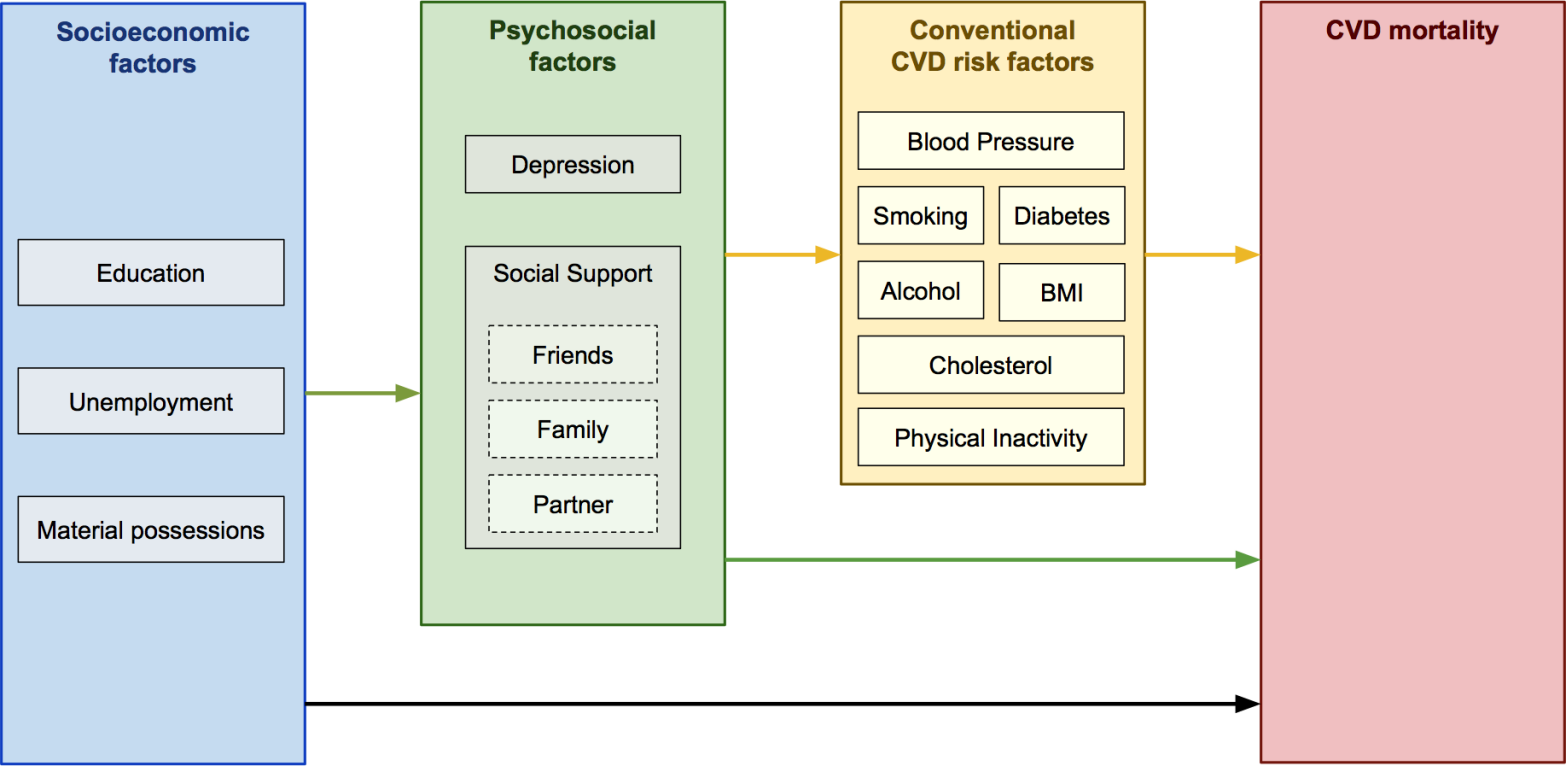
Theoretical direction of causal effects, from three sets of risk factors, to CVD mortality. BMI, body mass index; CVD, cardiovascular disease.

The aim of this multicentre cohort study was to investigate the extent to which psychosocial and socioeconomic factors are associated with CVD mortality in three Eastern European populations. We examined whether these risk factors are independently associated with CVD even after adjusting for each others’ effects and whether psychosocial and socioeconomic factors help to explain the high rates of CVD mortality in Russia when compared to similar populations with lower rates (Poland and the Czech Republic). In addition, we also explored whether depression and lack of social support might explain socioeconomic differences in CVD mortality (as measured by educational attainment, unemployment, or material possessions). These concepts are illustrated in our causal diagram (Fig 1).

## Methods

### Ethics statement

The study was approved by the University College London/University College London Hospital ethics committee and by the local ethics committee in each participating centre. All participants gave written informed consent.

### Rationale

The Health, Alcohol and Psychosocial factors In Eastern Europe (HAPIEE) study is a multicentre prospective cohort study of urban populations living in Russia, Poland, and the Czech Republic. The rationale for study was described in a cohort description published in 2006 [13]. Briefly, the study was designed to investigate the role of nutritional-, psychosocial-, and alcohol-related factors in the incidence of common disease (both at the within-country level, as well as to account for international differences in rates of disease). Previous publications from this study have reported associations between mortality and nutrition [14], alcohol consumption [15], and socioeconomic status [16]. Nutrition and alcohol did not explain why participants from Russia had twice the mortality rate when compared with participants from Poland or the Czech Republic. The current publication therefore assesses, in a systematic fashion, several original objectives of the study. We do not use data from the Lithuanian arm of the HAPIEE study as its baseline questionnaire did not include all the psychosocial variables of interest.

### Participants

Random population samples of 28,945 men and women aged 43–72 years at baseline during 2002–2005 were selected from population registers and electoral lists. The overall response rate was 59% (61% in Russia and Poland and 55% in the Czech Republic). We excluded 7,173 participants (25%) with a history of acute myocardial infarction, stroke, chronic heart disease, or angina, as well as those who scored positively on the Rose Angina Questionnaire.

Participants were linked to the national mortality registries using a national personal ID number in the Czech Republic and Poland. In Russia, linkage was done to the Novosibirsk Oblast regional mortality registry (which covers a land area larger than the Czech Republic) using surname, initials, and date of birth. Any potential inconsistencies were corrected manually, first by extracting the participant’s address and second (in case of inconclusive linkage) by telephoning the participant’s next of kin for verification. Nine hundred and five participants (4.2%) were lost to follow-up, primarily since they did not give consent to link their records with the national mortality registries, or due to migration (losses of 4%, 7%, and 1% in Czech, Polish, and Russian samples, respectively). This left an analytical sample of 20,867 participants. Follow-up was available to 31st December 2010 in Russia and Poland and 30th June 2013 in the Czech Republic (maximum follow up of 8.0, 8.9, and 11.3 years, respectively). The primary endpoint was CVD mortality (ICD-10 codes I00-99).

Of the participants who participated in wave 1, 66% also participants also participated in wave 2 of the HAPIEE study. This was collected in 2006–2008, i.e., after a mean interval of 3.6 (range 1 to 6) years. In this paper, data from wave 2 were only used to inform the predictor matrix when imputing missing data, and were not themselves used as exposures or outcomes.

### Socioeconomic factors

At baseline, participants completed an extensive structured questionnaire, and underwent a standardised nurse examination in a clinic. Highest educational qualification was grouped into three categories: primary or less, secondary, and tertiary. Assuming that the average participants in these groups were separated by around three and six years of additional schooling, respectively, we modelled a linear relationship between these three categories. For self-reported economic activity, participants were classified into three groups: economically active; retired and no longer working; and currently unemployed. Participants were also asked about long-term unemployment, with four options (never, up to 3 months total, up to 3–12 months total, more than 12 months total); responses were dichotomised, comparing those unemployed for more than 12 months against the rest.

Possession of 10 material amenities (microwave, video recorder, colour television, washing machine, dishwasher, freezer, camcorder, satellite TV, telephone, and mobile phone; each coded 0/1) was used to derive a standardised continuous scale. For current material deprivation, three questions asked about how often the subjects had difficulties with paying for food, clothes, or bills. Each item was scored 0–3 to yield a total score of 0–12. This was modelled per one standard deviation greater material deprivation. All standardisation procedures used the mean and SD obtained from all three cohorts in combination (as opposed to standardising, so that the standardised mean in each country is zero). Similar to current material deprivation, another scale of early-life material deprivation asked about the same items, this time with participants retrospectively recalling their childhood.

In addition, participants were asked whether the *‘Changes since 1989 have been good or bad for your general social position*?*’* Original responses in five categories (very good, good, no change, bad, very bad) were regrouped into three categories.

### Psychosocial factors

Social support is typically measured with a multifaceted instrument, covering aspects of friends, family, social clubs, and marital status. Given the larger power of our study, we did not combine these separate domains. For marital status, we compared the ‘married/cohabiting’ reference group against ‘divorced/widowed’, or ‘single’. *‘Are you a member of a club/organization*’ was kept binary. ‘*How often are you in contact with relatives outside of your household*?’ originally had six options: ‘*several times a week*’; ‘*about once a week*’; ‘*several times a month*’; ‘*about once a month*’; ‘*less than once a month*’; ‘*I don’t have any relatives*’. We dichotomised this at the *‘at least monthly’* threshold. A separate question, ‘*How often do you visit friends outside of your household*?’, was handled similarly.

Depressive symptoms were assessed with the CESD-20 questionnaire (range 0–60).[17] A binary trait for depressive symptoms was defined as CESD-20 score ≥ 16. Perceived control was assessed with a scale developed by the MacArthur programme on midlife development [18]. The subscale ‘control over life’ (range 0–40) was used as a standardised continuous variable.

### Conventional cardiovascular risk factors and other covariates

Covariates measured at baseline included country, age, gender, and 11 conventional CVD risk factors: smoking status (five categories of never smoker, ex-smoker; 1–10, 11–20, and 21+ cigarettes a day), diabetes; and physical activity (dichotomised at 2.5 hours a week) were determined by self-report. Clinical examination [13] determined systolic blood pressure (modelled linearly from 115 mmHg onwards [19]); as well as BMI and total and HDL serum cholesterol (whereby all three were modelled with linear and square terms after centring at 23 kg/m^2^, 6 mmol/L, and 1.5 mmol/L, respectively). Alcohol intake in the last 12 months was self-reported using the graduated frequency questionnaire (GFQ) [20]. The United Kingdom Chief Medical Officer’s alcohol guideline advises men and women to consume less than 14 units of alcohol (equivalent to 112 grams of ethanol) per week. Participants were categorised as either nondrinkers, drinking within these guidelines, drinking up to twice the guideline limit, or drinking more than twice the guideline limit. For completeness, we included three additional alcohol-related covariates. Frequency of alcohol consumption was dichotomised at the once a week threshold. A pattern of binge drinking was defined if men/women reported consuming more than 100 g/60 g of ethanol in one episode at least monthly. The CAGE questionnaire was used to evaluate symptoms of problems with alcohol and was dichotomised at 2 or above.

### Statistical analyses

#### Missing data

Between 0%–12% of the data was missing for each variable (Tables 1 and S1). This was imputed from 10 multiple imputation models that included vital status, follow-up time, and all covariates. The predictor matrix additionally included key variables that were associated with values for missing variables (where these were known), as well as their absence (where these were unknown), taken from either the baseline survey or a follow-up survey done three years later on the same participants. These predictors were further self-reported details about the time that participants spent on sports, games, and hiking; the tendency to rely only on self (as opposed to others); overcommitment at work; amount of trust in the local area; perceptions that money influences health; perceptions that others treat the participant unfairly; unemployment of others in the participant’s household; hypercholesterolaemia; antihypertensive drug and statin usage; and subsequent depression in wave 2.

**Table 1:** Baseline characteristics of the analytical sample.

	Total sample		CVD mortality		% missing and imputed
	n / mean	% / SD	n / mean	% / SD	
Participants	20,867	100%	556	2.7%	
Follow-up years (median, max)	7.2	11.3	4.4	10.0	
Age	57.2	7.03	62.0	6.20	0%
Male	9,700	46%	380	68%	0%
**Conventional CVD risk factors**					
Diabetes	1,554	7.4%	93	17%	0.1%
Smoking status:					0.6%
Never-smoker	9,941	48%	172	31%	
Occasional/past smoker	5,065	24%	133	24%	
Daily smoker, 1–10 cigarettes/day	2,074	9.9%	71	13%	
Daily smoker, 11–20 cigarettes/day	3,039	15%	142	25%	
Daily smoker, >20 cigarettes/day	747	3.6%	38	6.9%	
Blood pressure, systolic (mmHg)	138.9	22.5	153.2	25.8	9.2%
Total cholesterol (mmol/L)	5.95	1.25	6.06	1.44	10.5%
HDL cholesterol (mmol/L)	1.48	0.46	1.48	0.50	10.6%
Body mass index (kg/m^2^)	28.0	4.85	28.1	5.80	9.2%
Physically inactive	1,666	8.0%	100	18%	1.1%
Alcohol intake:					1.2%
Nil	4,033	19%	144	26%	
Up to UK guidelines	12,389	59%	274	49%	
Exceeding UK guidelines (1–2x over)	2,314	11%	65	12%	
Exceeding UK guidelines (>2x over)	2,131	10%	74	13%	
Alcohol drinking frequency:					1.4%
Nondrinker	3,942	19%	142	26%	
< once/week	10,534	50%	218	39%	
≥ once/week	6,391	31%	196	35%	
Binge drinking (≥ once/month)	2,705	13%	103	19%	1.4%
Possible problem drinking (CAGE ≥ 2)	1,408	6.7%	79	13%	8.1%
**Psychosocial factors**	* *				
Marital status:					0.2%
Married/cohabiting	15,713	75%	380	68%	
Divorced/widowed	4,257	20%	143	26%	
Single	897	4.3%	33	5.9%	
Social support					
Contacts relatives < once/month	4,966	24%	181	33%	0.4%
Contacts friends < once/month	7,554	36%	224	40%	0.6%
Not a member of a club	17,487	84%	491	88%	0.6%
Depression symptoms (possible case)	4,612	22%	161	29%	11.9%
Low perceived control (SD scale)	0.00	1.01	0.29	1.08	1.5%
**Socioeconomic factors**	* *				
Education					0.2%
Tertiary	5,263	25%	90	16%	
Secondary	13,459	65%	354	64%	
Primary	2,143	10%	112	20%	
Material possessions					
Low amenities, current (SD scale)	0.00	1.00	0.53	1.00	2.6%
Low amenities, early life (SD scale)	0.00	1.01	0.38	0.96	2.9%
Deprivation, current (SD scale)	0.00	1.02	0.22	1.16	0.9%
Deprivation, early life (SD scale)	0.00	0.99	0.05	1.11	1.0%
Unemployment, current	897	4.3%	28	5.0%	0.4%
Unemployment, long term	1,690	8.1%	44	7.8%	1.8%
Change in status since 1989:					0.9%
Improved	5,071	24%	103	19%	
Stayed the same	10,079	48%	261	47%	
Declined	5,718	27%	192	33%	

The sample shown is multiply imputed, combined across three countries and two genders. The final column shows the amount of missing/imputed data among the total sample (20,867 participants).

Abbreviations: HDL, high density lipoprotein; SD, standard deviation.

#### Main analysis

Cox regression was used to estimate hazard ratios (HRs) and 95% confidence intervals (CIs) for associations between psychosocial factors and mortality endpoints, using follow-up time as the time scale. Data were pooled across three cohorts and both genders. Three models were created with increasing levels of adjustment. Model 1 was adjusted for age, sex, and country. International differences in cardiovascular mortality in this region are known to be much larger in men than women. To examine potential causes of this, we looked at whether gender and/or being placed in Russia modifies the association between psychosocial/socioeconomic risk factors and CVD mortality. We included interactions that met a Bonferroni-adjusted *p*-value threshold of 0.05/14 = 0.0036. In model 2, we additionally adjusted for 11 conventional CVD risk factors for two reasons. First, as an indication of how much of the hazard from psychosocial/socioeconomic exposures might plausibly be mediated indirectly via conventional risk factors, and, second, as an indication of their direct effect size, through pathways not measured in this study. Model 3 was additionally adjusted for the six psychosocial and socioeconomic factors that are associated with CVD mortality (at *p*<0.05), following backwards-stepwise elimination from a larger model that began with all 14 candidate psychosocial factors. This approach was applied for three reasons: first, to help address statistical collinearity among the 14 exposures of interest; second, to identify a shortlist of top risk factors that might aetiologically lie on mutually exclusive pathways; and third, to identify a shortlist of top predictors that might be prioritised in future clinical care applications, such as novel risk prediction models. The proportional hazards assumption was tested using Schoenfeld residuals and checked graphically with log-log plots, with no evidence of its violation.

In all three models, we assumed no differences in the size of the risk factor–mortality associations between participants located in Poland, the Czech Republic, and Russia. There was insufficient power to explore heterogeneity between Poland and the Czech Republic, but we examined effect modification between participants from Russian versus Czech/Polish cohorts. Additional sensitivity analyses looked for effect modification by gender, repeating the main analysis after excluding imputed data, excluding participants with less than two years of follow-up (to reduce reverse causation bias), and using a similar 8-year follow-up for all three countries. In addition, we examined associations with all-cause mortality as the outcome instead of cardiovascular mortality.

#### Mediation analysis

Simple and complex models were compared with each other to evaluate the degree of attenuation and thereby infer approximate degree of mediation, using the formula:

Attenuation,oramountmediated=[log(FullyadjustedHR)−log(CrudeHR)]/[log(CrudeHR)].

For example, if the association between education and mortality is HR = 1.45 in model 1, and this attenuates to HR = 1.20 in model 2, then one can infer that the conventional cardiovascular risk factors (which are additionally included in model 2 but not in model 1) might account and potentially mediate around half of the pathway from education to mortality. Notably, this is a relatively crude method that is prone to differential measurement error as well as model mis-specification. Accordingly, we used this method only to provide very approximate and qualitative inferences about whether putative mediators are likely to play a small or large role, respectively.

#### Population Attributable Fraction

The Population Attributable Fraction denotes the proportion of mortality that could be prevented if the entire population were not exposed to a given risk factor (and assuming a causal relationship between exposure and outcome) [21]. In our study, it was calculated by fitting model 2 to the first set of imputed data and then using the *punafcc* package in STATA 14. As this package is unable to handle continuous risk factors, the continuous variable *material amenities* was dichotomised into two halves using the median as a cut off. For education, we calculated attribution if everybody without tertiary education would attain tertiary education. BMI was dichotomised as obese (BMI > 30 kg/m^2^) or not. This study is reported as per STROBE guidelines (S1 Checklist).

#### Analysis formulation

While the overall analyses we report correspond with the prespecified aims of the study, we did not have a detailed analysis plan prespecified. Strategic decisions about which analytic approach to use were mostly made before the analysis by applying methods that we have previously applied in other publications. For example, our mediation analysis has previously been applied to other publications from the HAPIEE and Whitehall II cohorts.[14, 22, 23] Our evaluation of population-attributable risk reflects the method we have previously applied for the IPD-Work consortium.[24] The a priori variables we included in models 1 and 2 reflect the approach we have taken with the MORGAM consortium [2]. In contrast to our previous publications, the current analysis investigates multiple collinear exposures. Our decision to use a stepwise approach to inform variable selection in model 3 was inspired by the use of this technique in the derivation of clinical risk prediction models [25]. Alcohol consumption was not initially included in the analysis (since our previous publication found that alcohol did not explain differences in rates of disease between cohorts), but alcohol-related covariates were later included in the analysis, at the request of peer reviewers.

## Results

Baseline characteristics of participants in the analytical sample are shown in **Table 1**. There were 556 deaths from CVD (249 out of 6,923 participants in Russia, 134 out of 7,039 in Poland, and 173 out of 6,905 in the Czech Republic). Participants who subsequently died of CVD had higher levels of most risk factors when compared to those who did not die of CVD. There was an interaction between sex and country: men in Russia had higher HRs than expected based on the sum of the ‘male’ and the ‘Russian’ indicator variables alone (HR for the interaction term = 1.77 [1.23–2.55], *p* = 0.002). This term was kept in all subsequent models.

### Models 1 and 2

**Fig 2:**
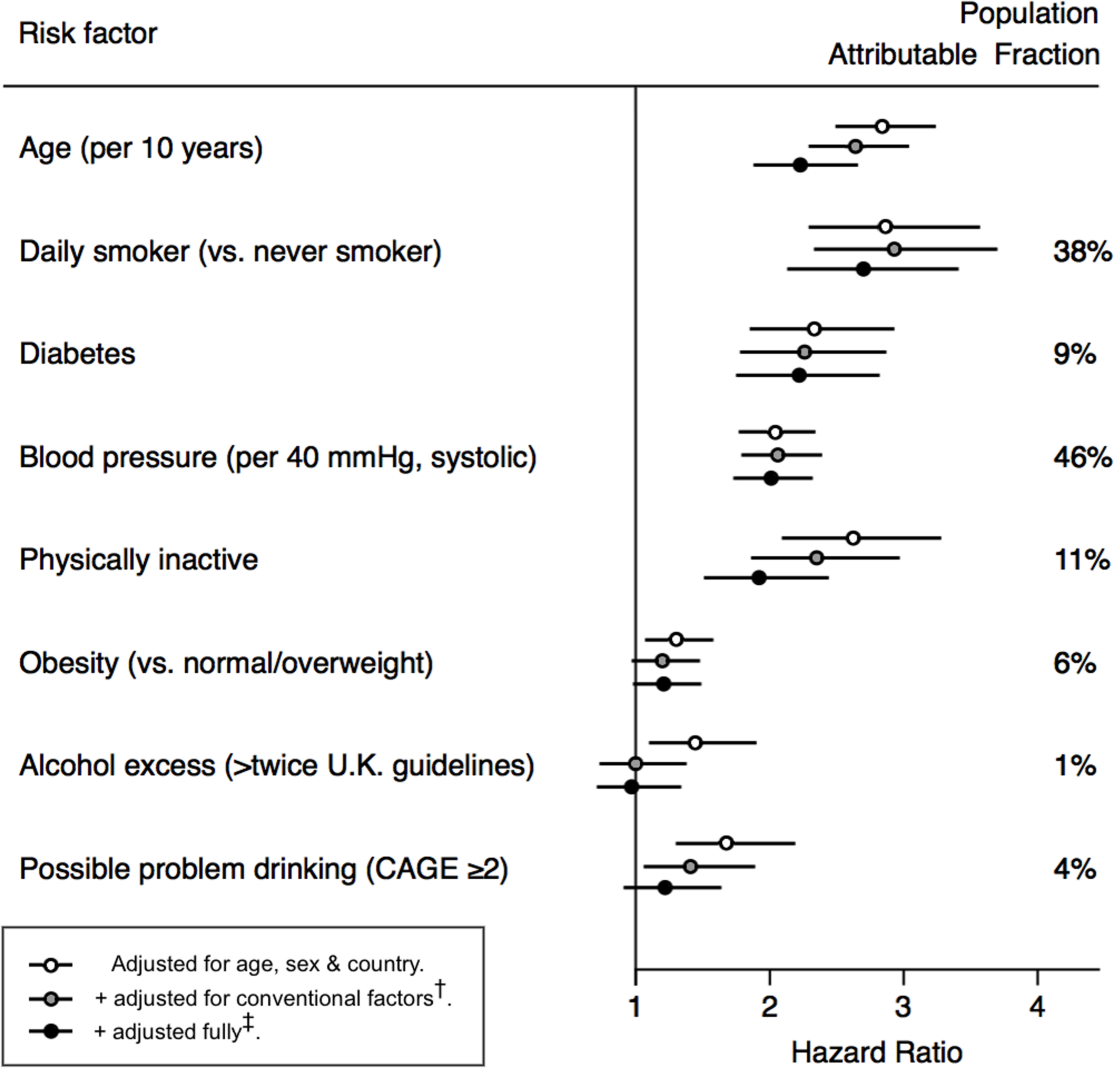
Associations of conventional cardiovascular risk factors with cardiovascular mortality. ^†^Conventional factors = diabetes, smoking, blood pressure, cholesterol, HDL, BMI, physical activity, alcohol. ^‡^Full adjustment = conventional factors + material possessions, depression, contacting relatives, contacting friends, friends*gender interaction, marital status, unemployment.

As expected, conventional CVD risk factors were associated with CVD mortality (Fig 2, S4 Table). Associations between 14 psychosocial and socioeconomic exposures and CVD mortality are shown in S5 Table. In model 1, 13 out of 14 psychosocial factors tested were associated with CVD mortality, with HRs ranging from 2.96 (1.97–4.46, *p* < 0.0001) for current unemployment to HR = 1.14 (1.05–1.23, *p* = 0.012) per one standard deviation increase in early life deprivation. Twelve associations remained significant after adjustment for eleven conventional CVD risk factors in model 2—this attenuated the remaining HRs by around a quarter.

Infrequent contact with friends was associated with outcomes more strongly in women than in men (Model 1 interaction with sex HR = 1.83 [1.26–2.66], *p* = 0.002, thus satisfying Bonferroni criteria). Model 3 therefore included both the conventional binary variable ‘*low friends*’ (which was handled similarly to the other five psychosocial factors) as well as the interaction term ‘gender*low friends’ (interactions were not used for the remaining five psychosocial and socioeconomic factors). We did not see any evidence of effect modification by country (S7 Table).

### Model 3

**Fig 3:**
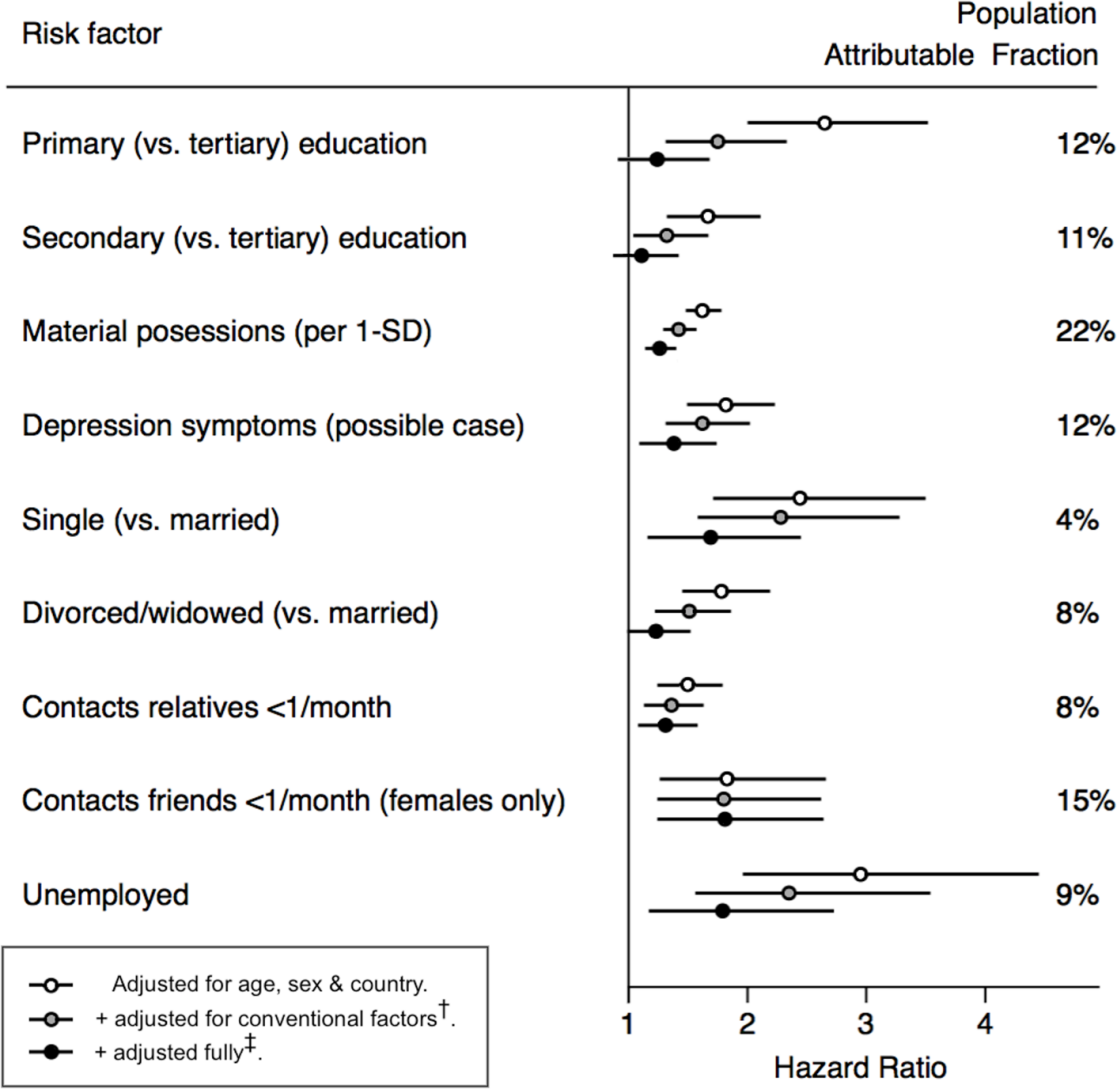
Associations of psychosocial and socioeconomic factors with cardiovascular mortality. SD, Standard Deviation ^†^Conventional factors = diabetes, smoking, blood pressure, cholesterol, HDL, BMI, physical activity, alcohol. ^‡^Full adjustment = conventional factors + material possessions, depression, contacting relatives, contacting friends, friends*gender interaction, marital status, unemployment.

Following full adjustment for other psychosocial and socioeconomic factors in model 3, six factors remained associated with the outcome (0.00001 < *p* < 0.007): *depression*, *low material amenities*, *current unemployment*, *infrequent contact with relatives*, *infrequent contact with friends (for female participants only)*, and *single marital status *(Fig 3). Following this full adjustment, the HR for education was largely attenuated in comparison with age-sex adjusted models. Consequently, we did not consider education to be one of the core socioeconomic variables in subsequent multivariate analyses. The PAFs in models adjusted for conventional risk factors ranged from around 8% [4%–13%] for infrequent contact with relatives to 22% [11%–31%] for low material amenities. Test of effect modification showed similar results in analyses stratified by gender or cohort (S6 and S7 Tables). Sensitivity analyses gave similar results when limiting follow-up time to eight years in all three countries, excluding those participants with less than two years of follow-up, excluding imputed data, when using all-cause mortality as the outcome (S8–S11 Tables), or when increasing the number of psychosocial/socioeconomic covariates (in model 3) from 6 to all 14 factors (model 4 from S5 Table). In the full model for all-cause mortality, evidence of independent associations additionally emerged for *no club membership* and *education* (S11 Table).

### Mediation analysis

**Fig 4:**
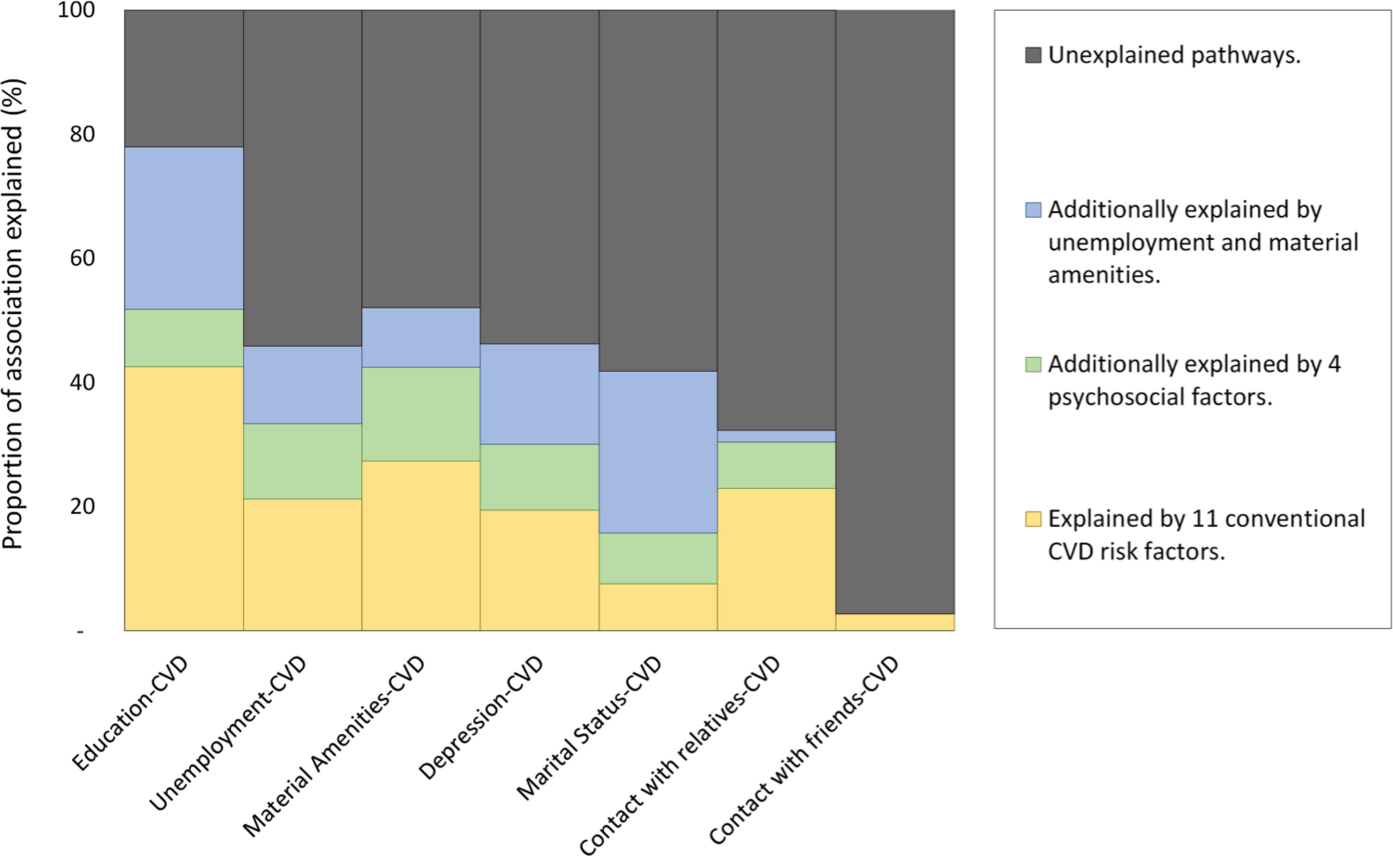
Attenuation among three socioeconomic (left side) and four psychosocial (right side) predictors of cardiovascular mortality. Created from four sequentially-adjusted models. The total height of each predictor on the y-axis (i.e., 100%) is equivalent to its association with cardiovascular mortality in model 1 (adjusted only for age, sex, and country). The yellow area represents the subsequent attenuation in association, following additional adjustment for 11 conventional CVD risk factors in model 2 (broadly corresponding to the yellow arrows in Fig 1). The green area represents the subsequent attenuation, following additional adjustment for four psychosocial variables (i.e., green arrow in Fig 1; coefficients shown in S12 Table). The blue area represents subsequent attenuation, following additional adjustment for two socioeconomic variables in model 3. The black area accounts for the unexplained part still present in model 3 (i.e., direct or nonmediated effects; black arrow in Fig 1). CVD, cardiovascular disease.

We examined to what degree the HRs associated with the psychosocial and socioeconomic factors attenuated following various adjustments (Fig 4 and S12 Table). Adjustment for 11 conventional risk factors attenuated these by about one quarter. Additional adjustment for other psychosocial and socioeconomic factors attenuated these by an additional quarter. As two exceptions, the HRs for marital status and limited contact with friends and relatives did not attenuate to this level. This suggests very little overlap between the potential effects of these facets of social support, and that conventional risk factors may play a negligible role in mediating any of these effects. Overall, our data suggests that depression and social support constructs, if causal, may operate independently to socioeconomic constructs, potentially along separate mechanistic pathways.

As few studies have reported to what degree the associations between alcohol and mortality attenuate after adjustment for psychosocial factors, we additionally report these here. There was little evidence that total alcohol consumption or binge drinking was associated with CVD mortality in models adjusted for conventional risk factors. However, people scoring positive on the CAGE screening questionnaire for possible problems with alcohol had 41% greater risk of CVD in models adjusted for age, sex, and country. This attenuated by around one half, following adjustment for six psychosocial and socioeconomic factors.

### International differences

**Fig 5:**
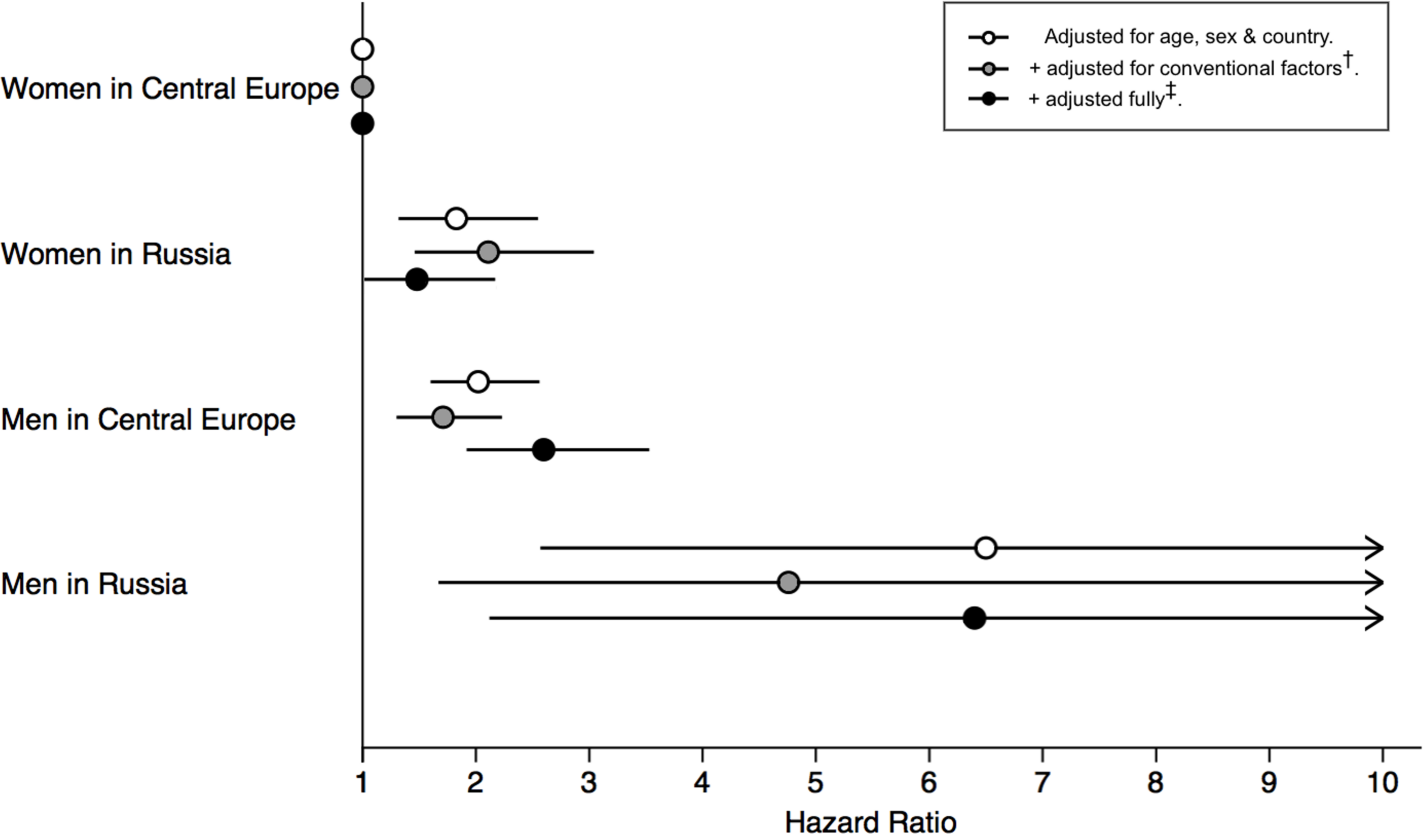
Increased risk of cardiovascular mortality from being male and/or being in Russia (versus being a female in Central Europe). Central Europe = Poland or Czech Republic. There were 109, 67, 198, and 175 events, respectively in the four groups shown (from top to bottom). ^†^Conventional factors = diabetes, smoking, blood pressure, cholesterol, HDL, BMI, physical activity, alcohol. ^‡^Full adjustment = conventional factors + material possessions, depression, contacting relatives, contacting friends, friends*gender interaction, marital status, unemployment.

CVD mortality risk was substantially higher in the Russian cohort. In men, the age-adjusted HR for being in Russia versus Central Europe was 2.86 [2.31–3.54]; this excess risk was not reduced following adjustment for conventional CVD risk factors (HR = 2.78 [2.15–3.59]) or following additional adjustment for psychosocial and socioeconomic factors (HR = 2.77 [2.13–3.61]) (Fig 5). In women, the HR of being in Russia versus Central Europe was 1.59 [1.15–2.19]; this difference was exacerbated following adjustment for conventional factors (HR = 2.15 [1.45–3.18]) but returned to a level comparable to crude models following additional adjustment for psychosocial and socioeconomic factors (HR = 1.64 [1.09–2.46]) (S13 Table).

## Discussion

This large prospective cohort study including adults from Russia, Poland, and the Czech Republic found independent associations between six psychosocial factors and subsequent cardiovascular mortality following full mutual adjustment. Depression and social support factors did not substantially attenuate socioeconomic gradients in CVD mortality, suggesting that these mechanisms are more likely to be complementary, as opposed to being mediatory. Contrary to our expectations, the twofold higher risk of CVD mortality seen in participants from Russia (when compared to participants from Poland or the Czech Republic) was not reduced following adjustment for conventional, psychosocial, and socioeconomic factors.

### Comparison with research in Western Europe and Northern America

Most of the associations we report are broadly consistent with prior studies, primarily from more affluent countries where all-cause mortality is often reported more commonly than our outcome of cardiovascular mortality.[2–5] However, we found current unemployment to be associated with an unusually high level of risk in our study (HR = 2.96 [1.97–4.46] for all-cause mortality, age-sex adjusted), which is more than twice the estimate from a recent meta-analysis (HR = 1.59 [1.42–1.77]) [3]. This strong effect may be plausible, however, if unemployment protections are weaker in Eastern Europe than elsewhere, which might highlight policy weaknesses for intervention.

Literature on material conditions has mostly focused on area-level measures of exposure [26, 27], while available studies at the individual level have often measured just 1–2 possessions, not aggregated such possessions into a summary score, or not controlled for blood pressure/cholesterol [28]. One comprehensive study in Russia did not find an association between material goods and/or amenities and all-cause mortality once education was controlled for [29]. Our larger study found the opposite pattern: that material amenities was the principal socioeconomic factor, which displaced education in multivariate analysis. Our analysis is consistent with prior reports of how education and material conditions may be measuring the same underlying socioeconomic construct, and it emphasises how material amenities might be a more sensitive socioeconomic predictor of cardiovascular mortality than education.

### Putative mechanisms via conventional CVD risk factors

Previous studies have suggested that conventional risk factors might account for around 30%–50% of the primary association between socioeconomic factors and mortality [2, 30, 31], consistent with our analysis of education. However, in our data the proportion explained by conventional factors was much smaller for social support factors, such as only 3% for lack of contact with friends and 8% for single marital status. Therefore, the role of conventional risk factors might be even smaller among psychosocial factors (such as social support and also depression) that have been less studied to date.

### Putative mechanisms via depression and social support

To our knowledge, this is the largest study to look at whether primary socioeconomic gradients in CVD can be attenuated following adjustment with psychosocial factors such as depression and social support. In Whitehall II, the occupational gradient in nonfatal coronary heart disease did not attenuate substantially following inclusion of social support measures [22], consistent with our findings. Instead, work-related stressors accounted for half of the occupational gradient in Whitehall II. We did not evaluate work factors given that a recent meta-analysis has shown their association with coronary heart disease to be comparatively small [24]. Other studies have typically used single-item instruments [32], or not assessed social support and conventional risk factors in the same study [30, 31]. Our study has confirmed relatively robustly that depression and social support do not account for much of socioeconomic gradients, even in a large cohort with high prevalence of exposure and outcome.

Our results suggest that the primary association between psychosocial factors (such as depression or social support) and CVD is unlikely to be mediated or confounded by conventional and other psychosocial risk factors, an area of limited prior study [33]. For example, elevated risk arising from exposure to one of the three dimensions of low social support was not offset by protection in another dimension, suggesting that cardiovascular health may be protected by some contact with friends, family, and a spouse. It appears that, in contrast to socioeconomic factors, each psychosocial factor is distinctly separate and does not relate to a common underlying construct. While attempts have been made to identify biomarkers of socioeconomic gradients, comparatively few have attempted to discover the mediators of psychosocial factors [23]. Our analysis suggests that depression is unlikely to be a major mediator of social support and socioeconomic pathways.

There is a paucity of studies investigating the potential mechanisms and mediators of psychosocial risk factors. We estimate, relatively crudely, that about one quarter of such hazards may be mediated by conventional CVD risk factors, and up to another quarter may come from other psychosocial factors such as depression. Measurement error in mediators biases our estimates of mediation towards the null, so future studies with time-varying mediators may be able to demonstrate larger proportions mediated [30]. In the case that such analyses fail to account entirely for the mechanistic pathways from psychosocial factors to CVD, additional hypotheses for investigations are warranted. These could include improved diet, or use of healthcare services (from better health knowledge, or fewer financial- and time-barriers in accessing care). Furthermore, direct molecular effects might be better clarified with emerging ‘omics technologies.

### International differences

We are not aware of other cohort studies that have used a standardised protocol to investigate why cardiovascular mortality is so dramatically high in Russia when directly compared to elsewhere. Ecological data from the Multinational MONItoring of trends and determinants in CArdiovascular disease (MONICA) study suggested that 0%–50% of the variations across time, in Europe and Russia, might be attributable to differences in conventional risk factors [7]. This is supported by studies of smoking and alcohol in Russia [34, 35]. Using different methods 30 years later, we find that conventional, psychosocial and socioeconomic factors do not account for much of the difference between Central European and Russian cohorts. We have previously shown that two other hypothesised factors, alcohol [15] and dietary factors [14], made only minor contributions to explaining the intercohort differences in mortality. After considering the results of the current study and the wider literature, we were unable to clarify the reasons for the very high cardiovascular mortality in Russian men. It is possible that international differences in access to healthcare, in healthcare seeking behaviour, or in the quality of care received, may help to clarify this question. Somewhat problematically, any such factors would need to have a large differential impact on male mortality but only a small differential impact on female mortality. Another area of enquiry might be gender norms and expectations. For example, women in Russia retire five years earlier than men. It is plausible that gender roles are more extremely polarised in such countries, which ultimately harms the health of men more than women [36].

### Strengths and limitations

Several limitations of this study should be considered when interpreting the results. First, these urban population samples are not necessarily representative of whole countries, as both exposures and mortality might be different in rural settings. Second, study participants may have been healthier than nonresponders, making us blind to what happens among those facing the greatest health and social problems. This would have led to underestimated HRs and population-attributable risk fractions. That said, our sample still detected considerable variation in cardiovascular mortality by country and socioeconomic status, as well as a considerable burden of psychological distress (e.g., 22% of participants screening positive for possible depression). Third, although we used predominantly well-established instruments to assess psychosocial exposures, their self-reported nature may be affected by response bias. For example, those who report adverse profiles might be more neurotic or less conscientious in their personality, which might instead be the underlying cause of our observed associations [37, 38]. Fourth, measurement error in mediators leads to underestimation in the degree of attenuation [39], and more importantly, any unmeasured confounders between mediators and outcome could have biased the results of our mediation analyses in either direction [40]. Fifth, our study makes no claims about causality due to its observational design. While some studies have supported a causal interpretation [41], the observational associations we report between CVD and social and psychosocial factors may be partly due to reverse causation or unmeasured confounding [42]. Sixth, it is uncertain how generalisable our results might be to other countries, given the social history and high mortality rates in this region.

As well as limitations, our study also has important strengths. First, this is the first prospective cohort study to our knowledge of psychosocial factors with a standardised methodology across multiple countries, where one country has twice the mortality rate of the others. Second, it is one of the largest prospective cohort studies that has assessed multiple psychosocial factors whilst concurrently controlling for all the conventional cardiovascular risk factors (including cholesterol and blood pressure). Third, most of the risk factors and covariates were measured using well-established and widely used psychosocial questionnaires and laboratory techniques.

### Conclusion

Psychosocial and socioeconomic factors are powerful predictors of cardiovascular mortality in Eastern Europe, similarly to elsewhere. For some risk factors, such as unemployment, these associations appear stronger than elsewhere, indicating potential areas for policy intervention. Surprisingly, most of the associations between psychosocial risk factors and CVD were attenuated by only a small amount when adjusted for each other. This suggests that there may be a lot of nuanced pathways which could be relatively independent of one another, although we are aware that the causality of these associations requires further confirmation. The massive burden of cardiovascular mortality seen in Russia remains largely unexplained.

## Supporting information

S1 Checklist STROBE guidelines, for reporting cohort studies. (DOCX)

S1 Table Baseline data, stratified by missing status. (DOCX)

S2 Table Baseline data, stratified by country. (DOCX)

S3 Table Baseline data, stratified by gender. (DOCX)

S4 Table Conventional risk factors and cardiovascular mortality. 556 events among 20,867 participants.
(DOCX)

S5 Table Psychosocial factors and cardiovascular mortality. Main analysis. 556 events among 20,867 participants.
(DOCX)

S6 Table Psychosocial factors and cardiovascular mortality: gender interactions. Hazards greater than one indicate a higher hazard in male participants, compared to female participants.
(DOCX)

S7 Table Psychosocial factors and cardiovascular mortality: country interactions. Hazards greater than one indicate a higher hazard in participants from Russia (versus Czech Republic/Poland).
(DOCX)

S8 Table Psychosocial factors and cardiovascular mortality: complete cases. Data shows associations for 326 events, among those with no missing data on any covariate (*N* = 13,727).
(DOCX)

S9 Table Psychosocial factors and cardiovascular mortality: excluding 2 years. Data shows associations for 458 events, among those with at least two years of follow-up (*N* = 20,560).
(DOCX)

S10 Table Psychosocial factors and cardiovascular mortality: limiting follow-up to eight years. Data shows associations for 514 events, among all participants (*N* = 20,867), whereby follow-up time was censored at 8.0 years in all three countries.
(DOCX)

S11 Table Psychosocial factors and all-cause mortality. 1,572 events among 20,867 participants.
(DOCX)

S12 Table Attenuation of psychosocial factors. Hazard ratios for cardiovascular mortality (in rows) attenuate from left to right, following the sequential addition of psychosocial covariates (columns). 556 events among 20,867 participants.
(DOCX)

S13 Table Attenuation of country-dummies. Hazard ratios for cardiovascular mortality from being in Russia (versus being in Central Europe, shown in rows) attenuates from left to right, following the sequential addition of psychosocial covariates (columns). 556 events among 20,867 participants. Results are stratified for men (380 events) and women (176 events).
(DOCX)

## Abbreviations

CI: confidence interval
CVD: cardiovascular disease
GFQ: graduated frequency questionnaire
HAPIEE: Health, Alcohol and Psychosocial factors In Eastern Europe
HR: hazard ratio
MONICA: Multinational MONItoring of trends and determinants in CArdiovascular disease
PAF: population attributable fraction
