# Supplementary material for: Psychosocial and socioeconomic determinants of cardiovascular mortality in Eastern Europe: A multicentre prospective cohort study
Source: PLoS Med. 2017 Dec 6;14(12):e1002459. doi: 10.1371/journal.pmed.1002459 (PMC5718419; doi:10.1371/journal.pmed.1002459)
Supplement: S1 Checklist — (DOCX) [file pmed.1002459.s001.docx]

**S1 Checklist. STROBE guidelines, for reporting cohort studies.**

| **Title and abstract** | | 1 | (*a*) Indicate the study’s design with a commonly used term in the title or the abstract  **Yes: *“Psychosocial and socioeconomic determinants of cardiovascular mortality in Eastern Europe: a multicentre prospective cohort study”*** |
| --- | --- | --- | --- |
|  |  |  | (*b*) Provide in the abstract an informative and balanced summary of what was done and what was found **Yes** |
| Introduction | | | |
| Background/rationale | | 2 | Explain the scientific background and rationale for the investigation being reported  **Yes: *“One weakness is that most evidence comes from the Western world, making it unclear to what extent these findings are generalizable.”*** |
| Objectives | | 3 | State specific objectives, including any prespecified hypotheses  **1) to investigate the extent to which socioeconomic and psychosocial factors associate with CVD mortality in four Eastern European populations. More specifically:**  **a) whether these two sets of risk factors are independently associated with CVD;**  **b) whether psychosocial factors accounted for socioeconomic differences in CVD mortality; and**  **c) whether socioeconomic and psychosocial factors help to explain the high rates of CVD mortality in in Russia when compared to populations with lower rates.** |
| Methods | | | |
| Study design | | 4 | Present key elements of study design early in the paper  **Yes under “Methods - Participants”** |
| Setting | | 5 | Describe the setting, locations, and relevant dates, including periods of recruitment, exposure, follow-up, and data collection  **Yes under “Methods - Participants”** |
| Participants | | 6 | (*a*) Give the eligibility criteria, and the sources and methods of selection of participants. Describe methods of follow-up  **Yes under “Methods - Participants”** |
|  |  |  | (*b*) For matched studies, give matching criteria and number of exposed and unexposed **n/a** |
| Variables | | 7 | Clearly define all outcomes, exposures, predictors, potential confounders, and effect modifiers. Give diagnostic criteria, if applicable  **Yes under “Methods - Socioeconomic factors” and “Methods - Psychosocial factors”** |
| Data sources/ measurement | | 8* | For each variable of interest, give sources of data and details of methods of assessment (measurement). Describe comparability of assessment methods if there is more than one group  **Yes under “Methods - Participants”** |
| Bias | | 9 | Describe any efforts to address potential sources of bias.  **Yes under “Methods - Statistical analysis” and under Results:**  **“*Sensitivity analyses gave similar results when limiting follow-up time to 8 years in all three countries; excluding those participants with less than 2 years of follow-up; excluding imputed data; when using all-cause mortality as the outcome; or when increasing the number of psychosocial/socioeconomic covariates (in model 3) from 6 to all 14 factors (S9-S12 Tables)”*.** |
| Study size | | 10 | Explain how the study size was arrived at  **Yes under “Methods - Participants”** |
| Quantitative variables | | 11 | Explain how quantitative variables were handled in the analyses. If applicable, describe which groupings were chosen and why  **Yes under “Methods”** |
| Statistical methods | | 12 | (*a*) Describe all statistical methods, including those used to control for confounding **yes** |
|  |  |  | (*b*) Describe any methods used to examine subgroups and interactions **yes** |
|  |  |  | (*c*) Explain how missing data were addressed  ***“Between 0-24% of the data was missing for each variable. This was imputed from 10 multiple imputation models that included vital status, follow-up time and all covariates.”* Sensitivity analyses excluded imputed data.** |
|  |  |  | (*d*) If applicable, explain how loss to follow-up was addressed.  **Yes under “Methods - Participants”** |
|  |  |  | (*e*) Describe any sensitivity analyses **yes** |
| Results | | | |
| Participants | 13* | (a) Report numbers of individuals at each stage of study—eg numbers potentially eligible, examined for eligibility, confirmed eligible, included in the study, completing follow-up, and analysed  **Yes under “Methods - Participants”** | |
|  |  | (b) Give reasons for non-participation at each stage  **Yes under “Methods - Participants”** | |
|  |  | (c) Consider use of a flow diagram **Not done, (as flow is quite simple),** | |
| Descriptive data | 14* | (a) Give characteristics of study participants (eg demographic, clinical, social) and information on exposures and potential confounders **Yes, table 1** | |
|  |  | (b) Indicate number of participants with missing data for each variable of interest **Yes, table 1** | |
|  |  | (c) Summarise follow-up time (eg, average and total amount), **yes Table 1 for averages. Totals are given in methods:**  ***“Follow up of 8, 9, 7, and 11 years respectively [in the four cohorts analysed]”*.** | |
| Outcome data | 15* | Report numbers of outcome events or summary measures over time  **Yes in abstract:**  ***“676 participants died from CVD”*** | |
| Main results | 16 | (*a*) Give unadjusted estimates and, if applicable, confounder-adjusted estimates and their precision (eg, 95% confidence interval). Make clear which confounders were adjusted for and why they were included **Yes Figures 3 and 5** | |
|  |  | (*b*) Report category boundaries when continuous variables were categorized  **Yes, boundaries are given in the Methods section.** | |
|  |  | (*c*) If relevant, consider translating estimates of relative risk into absolute risk for a meaningful time period.  **Deemed to be less relevant, given the large number of estimates presented. Absolute risk estimates are likely to be large.** | |
| Other analyses | 17 | Report other analyses done—eg analyses of subgroups and interactions, and sensitivity analyses **Yes, S9-S12 Tables.** | |
| Discussion | | | |
| Key results | 18 | Summarise key results with reference to study objectives **Yes 1^st^ paragraph of discussion.** | |
| Limitations | 19 | Discuss limitations of the study, taking into account sources of potential bias or imprecision. Discuss both direction and magnitude of any potential bias  **Yes under “Discussion - Strengths and limitations”.** | |
| Interpretation | 20 | Give a cautious overall interpretation of results considering objectives, limitations, multiplicity of analyses, results from similar studies, and other relevant evidence  **Yes under “Discussion - Conclusion”.** | |
| Generalisability | 21 | Discuss the generalisability (external validity) of the study results  **Not that applicable, since we are testing generalizability in unusual settings. Discussed “Discussion - Comparison with research in Western settings”.** | |
| Other information | | | |
| Funding | 22 | Give the source of funding and the role of the funders for the present study and, if applicable, for the original study on which the present article is based **Yes** | |

*Give information separately for exposed and unexposed groups.
