# Supplementary material for: Psychosocial and socioeconomic determinants of cardiovascular mortality in Eastern Europe: A multicentre prospective cohort study
Source: PLoS Med. 2017 Dec 6;14(12):e1002459. doi: 10.1371/journal.pmed.1002459 (PMC5718419; doi:10.1371/journal.pmed.1002459)
Supplement: S1 Table — (DOCX) [file pmed.1002459.s002.docx]

**S1 Table. Baseline data, stratified by missing status.**

|  | Participants with some missingness | Participants with no missingness | Amount of missingness | | | | |
| --- | --- | --- | --- | --- | --- | --- | --- |
|  |  |  | Czech Rep. | | Poland | | Russia |
| Participants | 7 140 (34%) | 13 727 (66%) |  | |  | |  |
| Follow-up years (median, max) | 7.1, 10.8 | 7.6, 11.3 |  | |  | |  |
| Events (CVD mortality) | 230 (3.2%) | 326 (2.4%) |  | |  | |  |
| ***Conventional risk factors*** |  |  |  | |  | |  |
| Age in years, mean (SD) | 57.7 (7.1) | 57.0 (7.0) | 0.0% | | 0.0% | | 0.0% |
| Male | 3 113 (44%) | 6 587 (48%) | 0.0% | | 0.0% | | 0.0% |
| Diabetes | 590 (8.3%) | 962 (7.0%) | 0.3% | | 0.2% | | 0.0% |
| Smoking Status: |  |  |  | |  | |  |
| Non-smoker | 3 466 (49%) | 6 480 (47%) |  | |  | |  |
| Occasional/Past smoker | 1 589 (22%) | 3 473 (25%) |  | |  | |  |
| Daily smoker, 1-10 cigarettes/day | 770 (11%) | 1 306 (9.5%) | 1.4% | | 0.3% | | 0.0% |
| Daily smoker, 11-20 cigarettes/day | 1 062 (15%) | 1 974 (14%) |  | |  | |  |
| Daily smoker, >20 cigarettes/day | 249 (3.5%) | 494 (3.6%) |  | |  | |  |
| Blood pressure, systolic (mmHg) | 139.7 (22.4) | 138.8 (21.8) | 16% | | 12% | | 0.3% |
| Cholesterol, total (mmol/L) | 6.00 (1.18) | 5.97 (1.16) | 20% | | 12% | | 0.4% |
| HDL (mmol/L) | 1.49 (0.55) | 1.47 (0.39) | 20% | | 12% | | 0.0% |
| Body Mass Index (kg/m^2^) | 28.2 (4.9) | 27.9 (4.7) | 16% | | 12% | | 0.0% |
| Physically inactive | 719 (11%) | 935 (6.8%) | 2.7% | | 0.7% | | 0.0% |
| Alcohol intake: |  |  |  | |  | |  |
| Nil | 2 380 (33%) | 1 792 (13%) | 2.2% | | 1.3% | | 0.0% |
| Up to UK guidelines | 3 482 (49%) | 8 818 (64%) |  |  |  |  |  |
| Exceeding UK guidelines (1-2x over) | 644 (9.0%) | 1 641 (12%) |  |  |  |  |  |
| Exceeding UK guidelines (>2x over) | 635 (8.9%) | 1 476 (11%) |  |  |  |  |  |
| Alcohol drinking frequency: |  |  |  | |  | |  |
| Non-drinker | 2 277 (32%) | 1 715 (12%) |  | |  | |  |
| < once/week | 3 087 (43%) | 7 425 (54%) | 3.5% | | 0.6% | | 0.0% |
| ≥ once/week | 1 776 (25%) | 4 588 (33%) |  | |  | |  |
| Binge drinking (≥1/month) | 790 (11%) | 1 912 (14%) | 3.5% | | 0.6% | | 0.0% |
| Possible problem drinking (CAGE ≥2) | 484 (6.8%) | 1 016 (7.4%) | 4.9% | | 19% | | 0.0% |
| ***Psychosocial factors*** |  |  |  | |  | |  |
| Marital Status: |  |  |  | |  | |  |
| Married/cohabiting | 5 155 (72%) | 10 558 (77%) |  | |  | |  |
| Divorced/widowed | 1 642 (23%) | 2 624 (19%) | 0.4% | | 0.2% | | 0.0% |
| Single | 343 (4.8%) | 545 (4.0%) |  | |  | |  |
| Social Support: |  |  |  | |  | |  |
| Contacts relatives <once/month | 1 707 (24%) | 2 875 (21%) | 0.8% | | 0.3% | | 0.0% |
| Contacts friends <once/month | 3 066 (43%) | 4 507 (33%) | 0.8% | | 0.7% | | 0.0% |
| Not a member of a club | 6 113 (86%) | 11 373 (83%) | 1.0% | | 0.8% | | 0.0% |
| Depression symptoms (possible case) | 1 719 (24%) | 2 728 (20%) | 6.1% | | 2.0% | | 27.7% |
| Low perceived control (SD scale) | 0.09 (1.02) | -0.05 (0.97) | 3.2% | | 1.4% | | 0.0% |
| ***Socioeconomic factors*** |  |  |  | |  | |  |
| Education |  |  |  | |  | |  |
| Tertiary | 1 527 (21%) | 3 739 (27%) |  | |  | |  |
| Secondary | 4 517 (63%) | 8 942 (65%) | 0.4% | | 0.1% | | 0.0% |
| Primary | 1 097 (15%) | 1 046 (7.6%) |  | |  | |  |
| Material possessions |  |  |  | |  | |  |
| Amenities, current (SD scale) | 0.12 (1.03) | -0.06 (0.98) | 6.0% | | 1.2% | | 0.5% |
| Amenities, early life (SD scale) | -0.08 (0.98) | 0.04 (1.00) | 5.1% | | 3.1% | | 0.6% |
| Deprivation, current (SD scale) | 0.15 (1.16) | -0.08 (0.93) | 1.5% | | 1.3% | | 0.0% |
| Deprivation, early life (SD scale) | 0.03 (1.04) | -0.01 (0.96) | 1.6% | | 1.0% | | 0.0% |
| Unemployment, current | 308 (4.3%) | 590 (4.3%) | 1.1% | | 0.2% | | 0.0% |
| Unemployment, long term | 561 (7.9%) | 1 135 (8.3%) | 0.9% | | 4.5% | | 0.0% |
| Change in status since 1989: |  |  |  | |  | |  |
| Improved a lot | 1 507 (21%) | 3 543 (26%) |  | |  | |  |
| Stayed the same | 3 463 (49%) | 6 615 (48%) | 2.0% | | 0.6% | | 0.0% |
| Declined | 2 170 (30%) | 3 569 (26%) |  |  | |  | |
