# Supplementary material for: Psychosocial and socioeconomic determinants of cardiovascular mortality in Eastern Europe: A multicentre prospective cohort study
Source: PLoS Med. 2017 Dec 6;14(12):e1002459. doi: 10.1371/journal.pmed.1002459 (PMC5718419; doi:10.1371/journal.pmed.1002459)
Supplement: S2 Table — (DOCX) [file pmed.1002459.s003.docx]

| **S2 Table. Baseline data,** **stratified by country.** | | | | | | | | |  |  |
| --- | --- | --- | --- | --- | --- | --- | --- | --- | --- | --- |
|  |  |  |  |  |  |  |  |  |  |  |
|  | Czech Republic | | Poland | | Russia | | | |  |  |
|  | n / mean | % / SD | n / mean | % / SD | n / mean | % / SD | | |  |  |
| Participants | 6 905 | 33% | 7 039 | 34% | 6 923 | 33% | | |  |  |
| Follow-up years (median, max) | 9.6, | 11.3 | 7.1, | 8.9 | 6.6, | 8.0 | | |  |  |
| Events (CVD mortality) | 173 | 31% | 134 | 24% | 249 | 45% | | |  |  |
| ***Conventional risk factors*** |  |  |  | | | | | |  |  |
| Age, mean (SD) | 57.6 | 7.1 | 56.8 | 7.0 | 57.3 | 7.0 | | |  |  |
| Male | 3 130 | 45% | 3 460 | 50% | 3 110 | 45% | | |  |  |
| Diabetes | 677 | 10% | 629 | 8.9% | 248 | 3.6% | | |  |  |
| Smoking Status: | | | | | | | |  |  |  |
| Non-smoker | 3 119 | 45% | 2 805 | 40% | 4 019 | 58% | | |  |  |
| Occasional/Past smoker | 2 088 | 30% | 2 051 | 29% | 926 | 13% | | |  |  |
| Daily smoker, 1-10 cigarettes/day | 746 | 11% | 649 | 9.2% | 679 | 10% | | |  |  |
| Daily smoker, 11-20 cigarettes/day | 824 | 12% | 1 167 | 17% | 1 049 | 15% | | |  |  |
| Daily smoker, >20 cigarettes/day | 129 | 1.9% | 368 | 5.2% | 250 | 3.6% | | |  |  |
| Blood pressure, systolic (mmHg) | 138.4 | 20.3 | 137.0 | 21.8 | 141.3 | 24.3 | | |  |  |
| Cholesterol, total (mmol/L) | 5.7 | 1.1 | 5.8 | 1.3 | 6.3 | 1.3 | | |  |  |
| HDL (mmol/L) | 1.4 | 0.4 | 1.5 | 0.4 | 1.6 | 0.5 | | |  |  |
| Body Mass Index (kg/m^2^) | 28.0 | 4.6 | 27.8 | 4.6 | 28.2 | 5.3 | | |  |  |
| Physically inactive | 827 | 12% | 572 | 8.1% | 269 | 3.9% | | |  |  |
| Alcohol intake: | | | | | | | | |  |  |
| Nil | 789 | 11% | 2 273 | 32% | 971 | 14% | | |  |  |
| Up to UK guidelines | 3 937 | 57% | 3 335 | 47% | 5 117 | 74% | | |  |  |
| Exceeding UK guidelines (1-2x over) | 1 137 | 16% | 670 | 9.5% | 507 | 7.3% | | |  |  |
| Exceeding UK guidelines (>2x over) | 1 042 | 15% | 760 | 11% | 328 | 4.7% | | |  |  |
| Alcohol drinking frequency: |  |  |  |  |  |  | | |  |  |
| Non-drinker | 802 | 12% | 2 206 | 31% | 934 | 13% | | |  |  |
| < once/week | 3 243 | 47% | 3 106 | 44% | 4 184 | 60% | | |  |  |
| ≥ once/week | 2 859 | 41% | 1 727 | 25% | 1 805 | 26% | | |  |  |
| Binge drinking (≥1/month) | 968 | 14% | 458 | 6.5% | 1 279 | 19% | | |  |  |
| Possible problem drinking (CAGE ≥2) | 366 | 5.3% | 348 | 5.0% | 694 | 10% | | |  |  |
| ***Psychosocial factors*** | | | | | | | | |  |  |
| Marital Status: | | | | | | | | |  |  |
| Married/cohabiting | 5 244 | 76% | 5 429 | 77% | 5 042 | 73% | | |  |  |
| Divorced/widowed | 1 469 | 21% | 1 206 | 17% | 1 586 | 23% | | |  |  |
| Single | 191 | 2.8% | 403 | 5.7% | 295 | 4.3% | | |  |  |
| Social Support: |  |  |  | | | | | |  |  |
| Contacts relatives <once/month | 757 | 11% | 2 192 | 31% | 2 008 | 29% | | |  |  |
| Contacts friends <once/month | 1 730 | 25% | 2646 | 38% | 3 186 | 46% | | |  |  |
| Not a member of a club | 4 992 | 72% | 6 167 | 88% | 6322 | 91% | | |  |  |
| Depression case | 1 253 | 18% | 1 642 | 23% | 1 715 | 25% | | |  |  |
| Low perceived control (SD scale) | 0.17 | 1.00 | -0.17 | 0.99 | 0.02 | 0.98 | | |  |  |
| ***Socioeconomic factors*** |  |  |  |  |  |  | | |  |  |
| Education |  |  |  |  | | | | |  |  |
| Tertiary | 1 016 | 15% | 2 206 | 31% | 2 042 | 29% | | |  |  |
| Secondary | 5 089 | 74% | 4 157 | 59% | 4 214 | 61% | | |  |  |
| Primary | 800 | 12% | 677 | 9.6% | 667 | 9.6% | | |  |  |
| Material possessions |  |  |  |  |  | | | |  |  |
| Low amenities, current (SD scale) | -0.23 | 1.00 | -0.07 | 0.98 | 0.30 | 0.95 | | |  |  |
| Low amenities, early life (SD scale ) | -0.48 | 0.75 | -0.09 | 1.03 | 0.54 | 0.93 | | |  |  |
| Deprivation, current (SD scale ) | -0.31 | 0.75 | 0.10 | 0.97 | 0.41 | 1.15 | | |  |  |
| Deprivation, early life (SD scale ) | 0.04 | 0.93 | -0.14 | 0.92 | 0.10 | 1.09 | | |  |  |
| Unemployment, current | 194 | 2.8% | 389 | 5.5% | 313 | 4.5% | | |  |  |
| Unemployment, long term | 398 | 5.8% | 562 | 8.0% | 735 | 11% | | |  |  |
| Change in status since 1989: |  |  |  |  | | | | |  |  |
| Improved a lot | 2 244 | 33% | 1 765 | 25% | 1 051 | 15% | | |  |  |
| Stayed the same | 3 695 | 54% | 3 274 | 47% | 3 113 | 45% | | |  |  |
| Declined | 965 | 14% | 1 997 | 28% | 2 758 | 40% | | |  |  |
| HDL=High Density Lipoproteins. SD=Standard Deviation. | | | | |  |  |  |  |  |  |
