# Supplementary material for: Psychosocial and socioeconomic determinants of cardiovascular mortality in Eastern Europe: A multicentre prospective cohort study
Source: PLoS Med. 2017 Dec 6;14(12):e1002459. doi: 10.1371/journal.pmed.1002459 (PMC5718419; doi:10.1371/journal.pmed.1002459)
Supplement: S3 Table — (DOCX) [file pmed.1002459.s004.docx]

**S3 Table. Baseline characteristics, stratified by gender.**

|  | Males | | Females | |  |  |
| --- | --- | --- | --- | --- | --- | --- |
|  | n / mean | % / SD | n / mean | % / SD |  |  |
| Participants | 9 700 | 47% | 11 167 | 54% |  |  |
| Follow-up years (median, max) | 7.1, | 11.3 | 7.6, | 11.3 |  |  |
| Events (CVD mortality) | 380 | 3.9% | 176 | 1.6% |  |  |
| ***Conventional risk factors*** |  |  |  |  |  |  |
| Age, mean (SD) | 57.4 | 7.0 | 57.0 | 7.0 |  |  |
| Diabetes | 795 | 8.2% | 759 | 6.8% |  |  |
| Smoking Status: |  |  |  |  |  |  |
| Non-smoker | 2 929 | 30% | 7 012 | 63% |  |  |
| Occasional/Past smoker | 3 128 | 32% | 1 937 | 17% |  |  |
| Daily smoker, 1-10 cigarettes/day | 914 | 9.4% | 1 160 | 10% |  |  |
| Daily smoker, 11-20 cigarettes/day | 2 100 | 22% | 9 39 | 8.4% |  |  |
| Daily smoker, >20 cigarettes/day | 629 | 6.5% | 118 | 1.1% |  |  |
| Blood pressure, systolic (mmHg) | 142.0 | 22.1 | 136.2 | 23.1 |  |  |
| Cholesterol, total (mmol/L) | 5.8 | 1.1 | 6.1 | 1.3 |  |  |
| HDL (mmol/L) | 1.4 | 0.46 | 1.6 | 0.44 |  |  |
| Body Mass Index (kg/m^2^) | 27.3 | 4.23 | 28.5 | 5.4 |  |  |
| Physically inactive | 1 050 | 11% | 615 | 5.5% |  |  |
| Alcohol intake: |  |  |  |  |  |  |
| Nil | 1 284 | 13% | 2 749 | 25% |  |  |
| Up to UK guidelines | 4 769 | 49% | 7 621 | 68% |  |  |
| Exceeding UK guidelines (1-2x over) | 1 779 | 18% | 535 | 4.8% |  |  |
| Exceeding UK guidelines (>2x over) | 1 869 | 19% | 261 | 2.3% |  |  |
| Alcohol drinking frequency: |  |  |  |  |  |  |
| Non-drinker | 1 228 | 13% | 2 715 | 24% |  |  |
| < once/week | 3 765 | 39% | 6 769 | 61% |  |  |
| ≥ once/week | 4 707 | 49% | 1 683 | 15% |  |  |
| Binge drinking (≥1/month) | 1 949 | 20% | 757 | 6.8% |  |  |
| Possible problem drinking (CAGE ≥2) | 1 244 | 13% | 164 | 1.5% |  |  |
| ***Psychosocial factors*** |  |  |  |  |  |  |
| Marital Status: |  |  |  |  |  |  |
| Married/cohabiting | 8 390 | 86% | 7 326 | 66% |  |  |
| Divorced/widowed | 986 | 10% | 3 279 | 29% |  |  |
| Single | 324 | 3.3% | 564 | 5.1% |  |  |
| Social Support: |  |  |  |  |  |  |
| Contacts relatives <once/month | 2 577 | 27% | 2 381 | 21% |  |  |
| Contacts friends <once/month | 3 653 | 38% | 3 910 | 35% |  |  |
| Not a member of a club | 7 849 | 81% | 9 632 | 86% |  |  |
| Depression case | 1 469 | 15% | 3 141 | 28% |  |  |
| Low perceived control (SD scale) | -0.09 | 0.99 | 0.08 | 1.01 |  |  |
| ***Socioeconomic factors*** |  |  |  |  |  |  |
| Education |  |  |  |  |  |  |
| Tertiary | 2 690 | 28% | 2 574 | 23% |  |  |
| Secondary | 6 221 | 64% | 7 238 | 65% |  |  |
| Primary | 789 | 8.1% | 1 355 | 12% |  |  |
| Material possessions |  |  |  |  |  |  |
| Low amenities, current (SD scale) | -0.13 | 1.00 | 0.11 | 1.00 |  |  |
| Low amenities, early life (SD scale ) | 0.02 | 1.02 | -0.02 | 1.01 |  |  |
| Deprivation, current (SD scale ) | -0.13 | 0.97 | 0.11 | 1.05 |  |  |
| Deprivation, early life (SD scale ) | -0.12 | 0.88 | 0.10 | 1.07 |  |  |
| Unemployment, current | 518 | 5.3% | 378 | 3.4% |  |  |
| Unemployment, long term | 718 | 7.4% | 978 | 8.8% |  |  |
| Change in status since 1989: |  |  |  |  |  |  |
| Improved a lot | 2 522 | 26% | 2 539 | 23% |  |  |
| Stayed the same | 4 554 | 47% | 5 530 | 50% |  |  |
| Declined | 2 624 | 27% | 3 097 | 28% |  |  |
| HDL=High Density Lipoproteins. SD=Standard Deviation. | | | | |  |  |
