# Supplementary material for: Psychosocial and socioeconomic determinants of cardiovascular mortality in Eastern Europe: A multicentre prospective cohort study
Source: PLoS Med. 2017 Dec 6;14(12):e1002459. doi: 10.1371/journal.pmed.1002459 (PMC5718419; doi:10.1371/journal.pmed.1002459)
Supplement: S4 Table — 556 events among 20,867 participants. (DOCX) [file pmed.1002459.s005.docx]

| **S4 Table. Conventional risk factors and cardiovascular mortality.**  556 events among 20,867 participants. | | | |  |
| --- | --- | --- | --- | --- |
|  | Hazard Ratios (95% confidence interval) | | |  |
|  | Model 1*^a^* | Model 2*^b^* | Model 3*^c^* |  |
| Age, per 10 years | **2.84 (2.49-3.24)** | **2.64 (2.29-3.04)** | **2.22 (1.87-2.65)** |  |
| Male gender | **2.03 (1.61-2.57)** | **1.71 (1.30-2.23)** | **2.59 (1.91-3.52)** |  |
| Russia (vs. Central European country) | **1.81 (1.30-2.53)** | **2.11 (1.46-3.04)** | **1.48 (1.01-2.17)** |  |
| Male-by-Russia interaction | **1.77 (1.23-2.55)** | 1.32 (0.88-1.97) | **1.67 (1.10-2.53)** |  |
| Smoking Status: |  |  |  |  |
| Non-smoker | 1 | **1** | 1 |  |
| Occasional/Past smoker | **1.50 (1.17-1.92)** | **1.57 (1.22-2.02)** | **1.58 (1.23-2.04)** |  |
| Daily smoker, 1-10 cigarettes/day | **2.37 (1.78-3.17)** | **2.50 (1.85-3.36)** | **2.33 (1.73-3.15)** |  |
| Daily smoker, 11-20 cigarettes/day | **3.04 (2.36-3.92)** | **3.15 (2.42-4.10)** | **2.86 (2.19-3.74)** |  |
| Daily smoker, >20 cigarettes/day | **4.03 (2.75-5.89)** | **3.75 (2.54-5.53)** | **3.46 (2.34-5.10)** |  |
| Blood pressure, systolic (per 40 mmHg) | **2.04 (1.77-2.34)** | **2.06 (1.79-2.39)** | **2.01 (1.73-2.32)** |  |
| Diabetes | **2.33 (1.85-2.93)** | **2.26 (1.78-2.87)** | **2.22 (1.75-2.82)** |  |
| Cholesterol, total (linear term, per 1 mmol/L) | 1.01 (0.93-1.09) | 1.00 (0.93-1.09) | 1.02 (0.94-1.10) |  |
| Cholesterol, total (quadratic term) | **1.03 (1.01-1.04)** | **1.03 (1.01-1.04)** | **1.03 (1.01-1.04)** |  |
| HDL (linear term, per 1 mmol/L) | 1.11 (0.87-1.41) | 1.27 (0.98-1.66) | 1.27 (0.98-1.64) |  |
| HDL (quadratic term) | 0.99 (0.95-1.03) | 0.96 (0.92-1.01) | 0.96 (0.92-1.01) |  |
| Body Mass Index (linear term, per 1 kg/m^2^) | **1.18 (1.12-1.25)** | **1.14 (1.07-1.20)** | **1.12 (1.05-1.18)** |  |
| Body Mass Index (quadratic term) | **1.00 (1.00-1.01)** | **1.00 (1.00-1.01)** | **1.00 (1.00-1.00)** |  |
| Physically inactive | **2.62 (2.09-3.28)** | **2.35 (1.86-2.97)** | **1.92 (1.51-2.44)** |  |
| Alcohol intake: |  |  |  |  |
| Nil | **1.81 (1.46-2.23)** | 1.06 (0.57-1.95) | 1.06 (0.58-1.93) |  |
| Up to UK guidelines | 1 | 1 | 1 |  |
| Exceeding UK guidelines (1-2x over) | 1.05 (0.79-1.39) | 0.88 (0.65-1.19) | 0.90 (0.66-1.22) |  |
| Exceeding UK guidelines (>2x over) | **1.44 (1.10-1.90)** | 1.00 (0.73-1.38) | 0.97 (0.71-1.34) |  |
| Alcohol drinking frequency: |  |  |  |  |
| Non-drinker | **1.87 (1.50-2.32)** | 1.70 (0.91-3.18) | 1.51 (0.81-2.80) |  |
| < once/week | 1 | 1 | 1 |  |
| ≥ once/week | 1.22 (0.99-1.49) | 1.05 (0.82-1.33) | 1.10 (0.87-1.40) |  |
| Binge drinking (≥1/month) | **1.32 (1.05-1.66)** | 1.08 (0.83-1.40) | 1.00 (0.77-1.30) |  |
| Possible problem drinking (CAGE ≥2) | **1.68 (1.30-2.19)** | **1.41 (1.06-1.89)** | 1.22 (0.91-1.64) |  |
| *^a^ Adjusted for Age, sex, country, male*Russian interaction* | | | |  |
| *^b^ Adjusted for Age; sex; country; male*Russian interaction; diabetes; smoking; blood pressure; cholesterol; HDL; BMI; physical activity; alcohol intake, frequency, binge pattern and problems.* | | | | |
| *^c^ Adjusted for Age; sex; country; male*Russian interaction; diabetes; smoking; blood pressure; cholesterol; HDL; BMI; physical activity; alcohol intake, frequency, binge pattern and problems;   marital status; seeing relatives; seeing friends; friends*gender interaction; depression; material amenities; current unemployment.* | | | | |
|  |  |  |  |  |
