# Supplementary material for: Psychosocial and socioeconomic determinants of cardiovascular mortality in Eastern Europe: A multicentre prospective cohort study
Source: PLoS Med. 2017 Dec 6;14(12):e1002459. doi: 10.1371/journal.pmed.1002459 (PMC5718419; doi:10.1371/journal.pmed.1002459)
Supplement: S5 Table — Main analysis. 556 events among 20,867 participants. (DOCX) [file pmed.1002459.s006.docx]

| **S5 Table. Psychosocial factors and cardiovascular mortality.**  Main analysis. 556 events among 20,867 participants. | | | | | |
| --- | --- | --- | --- | --- | --- |
|  |  |  |  |  |  |
|  | Hazard Ratios (95% confidence interval) | | | Model 4^d^ | Population Attributable |
|  | Model 1*^a^* | Model 2*^b^* | Model 3^c^ |  | Fraction (Model 2) |
| *Psychosocial factors* |  |  |  |  |  |
| Marital Status: |  |  |  |  |  |
| Married/cohabiting | 1 | 1 | 1 | 1 | 12 (8…16)% |
| Divorced/widowed | **1.78 (1.45-2.19)** | **1.51 (1.22-1.86)** | 1.23 (0.99-1.52) | **1.24 (1.00-1.55)** |  |
| Single | **2.44 (1.71-3.50)** | **2.28 (1.58-3.28)** | **1.68 (1.15-2.44)** | **1.72 (1.17-2.52)** |  |
| Social Support |  |  |  |  |  |
| Contacts relatives <once/month | **1.49 (1.24-1.79)** | **1.36 (1.13-1.63)** | **1.31 (1.08-1.58)** | **1.34 (1.10-1.63)** | 8 (4…13)% |
| Contacts friends <once/month | 0.85 (0.69-1.05) | 0.84 (0.68-1.03) | **0.74 (0.59-0.92)** | **0.72 (0.57-0.90)** |  |
| friends*female interaction | **1.83 (1.26-2.66)** | **1.80 (1.24-2.62)** | **1.81 (1.24-2.64)** | **1.87 (1.27-2.76)** | 15 (6…22)% |
| Not a member of a club | **1.62 (1.24-2.12)** | **1.33 (1.01-1.74)** | 1.24 (0.94-1.63) | 1.23 (0.93-1.63) |  |
| Depression case | **1.82 (1.49-2.23)** | **1.62 (1.31-2.02)** | **1.38 (1.09-1.74)** | **1.33 (1.04-1.69)** | 12 (9...15)% |
| Low perceived control (per 1-SD) | **1.30 (1.19-1.41)** | **1.19 (1.09-1.29)** | 1.04 (0.94-1.14) | 1.05 (0.95-1.16) |  |
| *Socioeconomic factors* |  |  |  |  |  |
| Education (per 3 fewer years) | **1.63 (1.41-1.87)** | **1.32 (1.14-1.53)** | 1.11 (0.96-1.29) | 1.14 (0.97-1.33) | 23 (8..36)% |
| Tertiary | 1 | 1 | 1 | 1 |  |
| Secondary | **1.67 (1.32-2.11)** | **1.32 (1.04-1.67)** | 1.11 (0.87-1.42) | 1.14 (0.89-1.46) |  |
| Primary | **2.65 (2.00-3.52)** | **1.75 (1.31-2.33)** | 1.24 (0.91-1.68) | 1.30 (0.94-1.78) |  |
| Material possessions |  |  |  |  |  |
| Amenities, current (per 1-SD) | **1.62 (1.48-1.78)** | **1.42 (1.29-1.57)** | **1.26 (1.14-1.40)** | **1.23 (1.09-1.37)** | 22 (11..31)% |
| Amenities, early life (per 1-SD) | 1.01 (0.90-1.13) | 1.00 (0.90-1.12) | 0.96 (0.85-1.07) | 0.95 (0.84-1.07) |  |
| Deprivation, current (per 1-SD) | **1.23 (1.13-1.33)** | **1.14 (1.05-1.23)** | 0.98 (0.89-1.07) | 0.95 (0.98-1.18) |  |
| Deprivation, early life (per 1-SD) | **1.14 (1.05-1.23)** | **1.11 (1.02-1.21)** | 1.06 (0.97-1.15) | 1.07 (0.98-1.18) |  |
| Unemployment, current | **2.96 (1.97-4.46)** | **2.35 (1.56-3.54)** | **1.80 (1.18-2.74)** | **1.75 (1.09-2.83)** | 9 (6..11)% |
| Unemployment, long term | **1.76 (1.27-2.42)** | **1.48 (1.06-2.06)** | 1.10 (0.75-1.59) | 1.14 (0.78-1.67) |  |
| Improvement in status since 1989 | 1 | 1 | 1 | 1 |  |
| No change in status since 1989 | 1.14 (0.91-1.44) | 1.02 (0.81-1.29) | 0.86 (0.68-1.09) | 0.84 (0.66-1.07) |  |
| Loss of status since 1989 | **1.36 (1.06-1.76)** | 1.17 (0.91-1.50) | 0.84 (0.65-1.10) | 0.84 (0.64-1.11) |  |
| *^a^ Adjusted for Age, sex, country, male*Russian interaction* | | | |  |  |
| *^b^ Adjusted for Age; sex; country; male*Russian interaction; diabetes; smoking; blood pressure; cholesterol; HDL; BMI; physical activity; alcohol intake, frequency, binge pattern and problems.* | | | | | |
| *^c^ Adjusted for Age; sex; country; male*Russian interaction; diabetes; smoking; blood pressure; cholesterol; HDL; BMI; physical activity; alcohol intake, frequency, binge pattern and problems;   marital status; seeing relatives; seeing friends; friends*gender interaction; depression; material amenities; current unemployment.*  *^d^ as in Model 3^c^, and additionally adjusted for 8 psychosocial and socioeconomic variables which did not reach conventional levels of statistical significance (club membership, percieved control, education, early life amenities, current deprivation, early life deprivation, long term unemployment, change in status since 1989).* | | | | | |
|  |  |  |  |  |  |
