# Supplementary material for: Psychosocial and socioeconomic determinants of cardiovascular mortality in Eastern Europe: A multicentre prospective cohort study
Source: PLoS Med. 2017 Dec 6;14(12):e1002459. doi: 10.1371/journal.pmed.1002459 (PMC5718419; doi:10.1371/journal.pmed.1002459)
Supplement: S6 Table — Hazards greater than one indicate a higher hazard in male participants, compared to female participants. (DOCX) [file pmed.1002459.s007.docx]

| **S6 Table. Psychosocial factors and cardiovascular mortality****: gender interactions.**  Hazards greater than one indicate a higher hazard in male participants, compared to female participants. | | | |
| --- | --- | --- | --- |
|  |  |  |  |
|  |  |  |  |
|  | Hazard Ratio (95% confidence interval) | | |
|  | Model 1*^a^* | Model 2*^b^* | Model 3*^c^* |
| *Psychosocial factors* |  |  |  |
| Marital Status: |  |  |  |
| Married/cohabiting | 1 | 1 | 1 |
| Divorced/widowed | 1.20 (0.80-1.81) | 1.16 (0.77-1.76) | 1.03 (0.68-1.55) |
| Single | 1.17 (0.56-2.44) | 1.10 (0.52-2.32) | 0.91 (0.43-1.92) |
| Social Support |  |  |  |
| Contacts relatives <once/month | 0.88 (0.59-1.30) | 0.92 (0.62-1.36) | 1.09 (0.73-1.65) |
| **Contacts friends <once/month** | **0.55 (0.38-0.80)*** | **0.56 (0.38-0.81)*** | **0.55 (0.38-0.81)*** |
| Not a member of a club | 1.07 (0.60-1.90) | 1.00 (0.56-1.80) | 1.06 (0.59-1.91) |
| Depression case | 1.24 (0.80-1.90) | 1.20 (0.78-1.84) | 1.21 (0.78-1.89) |
| Low perceived control (per 1-SD) | 0.87 (0.73-1.05) | 0.88 (0.73-1.05) | 0.87 (0.72-1.04) |
| *Socioeconomic factors* |  |  |  |
| Education |  |  |  |
| Tertiary | 1 | 1 | 1 |
| Secondary | 0.95 (0.55-1.65) | 0.92 (0.53-1.60) | 0.89 (0.51-1.55) |
| Primary | 0.76 (0.41-1.43) | 0.80 (0.43-1.51) | 0.79 (0.42-1.48) |
| Material possessions |  |  |  |
| Amenities, current (per 1-SD) | 0.81 (0.66-1.00) | 0.83 (0.67-1.02) | 0.84 (0.68-1.03) |
| Amenities, early life (per 1-SD) | 1.02 (0.82-1.27) | 1.08 (0.86-1.34) | 1.12 (0.90-1.40) |
| Deprivation, current (per 1-SD) | 1.01 (0.85-1.20) | 1.01 (0.85-1.20) | 1.01 (0.85-1.20) |
| Deprivation, early life (per 1-SD) | 1.07 (0.90-1.27) | 1.04 (0.88-1.23) | 1.03 (0.87-1.22) |
| Unemployment, current | 0.78 (0.27-2.21) | 0.69 (0.24-1.96) | 0.61 (0.21-1.71) |
| Unemployment, long term | 1.91 (0.92-3.97) | 1.70 (0.81-3.54) | 1.38 (0.65-2.92) |
| No change in status since 1989 | 0.87 (0.52-1.47) | 0.88 (0.52-1.47) | 0.90 (0.53-1.51) |
| Loss of status since 1989 | 0.88 (0.50-1.54) | 0.90 (0.51-1.56) | 1.00 (0.57-1.76) |
| **Age** | **0.96 (0.93-0.99)** | 0.97 (0.94-1.00) | 0.98 (0.95-1.01) |
| **Russia (vs. Central Europe)** | **1.77 (1.23-2.55)*** | 1.32 (0.88-1.97) | **1.67 (1.10-2.53)** |
| *^a^ Adjusted for Age, sex, country, male*Russian interaction* | | | |
| *^b^ Adjusted for Age; sex; country; male*Russian interaction; diabetes; smoking; blood pressure; cholesterol; HDL; BMI; physical activity;*  *alcohol intake, frequency, binge pattern and problems.* | | | |
| *^c^ Adjusted for Age; sex; country; male*Russian interaction; diabetes; smoking; blood pressure; cholesterol; HDL; BMI; physical activity;*  *alcohol intake, frequency, binge pattern and problems; marital status; seeing relatives; seeing friends; friends*gender interaction; depression; material amenities; current unemployment.*  ** P value < 0.0036 (i.e. Bonferroni adjustment of 0.05/14).* | | | |
|  | | | |
