# Supplementary material for: Psychosocial and socioeconomic determinants of cardiovascular mortality in Eastern Europe: A multicentre prospective cohort study
Source: PLoS Med. 2017 Dec 6;14(12):e1002459. doi: 10.1371/journal.pmed.1002459 (PMC5718419; doi:10.1371/journal.pmed.1002459)
Supplement: S7 Table — Hazards greater than one indicate a higher hazard in participants from Russia (versus Czech Republic/Poland). (DOCX) [file pmed.1002459.s008.docx]

| **S7 Table. Psychosocial factors and cardiovascular mortality: country interactions.**  Hazards greater than one indicate a higher hazard in participants from Russia (versus. Czech Republic/Poland). | | | | | |
| --- | --- | --- | --- | --- | --- |
|  |  |  |  |  |  |
|  |  |  |  |  |  |
|  | Hazard Ratio (95% confidence interval) | | |  |  |
|  | Model 1*^a^* | Model 2*^b^* | Model 3*^c^* | |  |
| *Psychosocial factors* |  |  |  | |  |
| Marital Status: |  |  |  | |  |
| Married/cohabiting | 1 | 1 | 1 | |  |
| Divorced/widowed | 1.18 (0.78-1.79) | 1.25 (0.83-1.89) | 1.20 (0.80-1.81) | |  |
| Single | 0.98 (0.47-2.03) | 1.12 (0.54-2.34) | 1.22 (0.58-2.55) | |  |
| Social Support |  |  |  | |  |
| Contacts relatives <once/month | 0.88 (0.59-1.23) | 0.95 (0.66-1.38) | 0.96 (0.66-1.39) | |  |
| Contacts friends <once/month | 1.18 (0.82-1.68) | 1.31 (0.92-1.87) | 1.28 (0.90-1.85) | |  |
| Not a member of a club | 1.39 (0.69-2.81) | 1.41 (0.69-2.86) | 1.37 (0.67-2.78) | |  |
| Depression case | 0.91 (0.63-1.33) | 0.91 (0.62-1.33) | 0.91 (0.61-1.34) | |  |
| Low perceived control (per 1-SD) | 0.86 (0.73-1.02) | 0.93 (0.79-1.10) | 0.92 (0.78-1.09) | |  |
| *Socioeconomic factors* |  |  |  | |  |
| Education |  |  |  | |  |
| Tertiary | 1 | 1 | 1 | |  |
| Secondary | 1.16 (0.73-1.86) | 1.21 (0.76-1.93) | 1.32 (0.83-2.12) | |  |
| Primary | 0.80 (0.46-1.41) | 0.88 (0.50-1.55) | 1.04 (0.59-1.83) | |  |
| Material possessions |  |  |  | |  |
| Amenities, current (per 1-SD) | 1.02 (0.84-1.23) | 1.01 (0.84-1.22) | 1.02 (0.85-1.23) | |  |
| Amenities, early life (per 1-SD) | 0.88 (0.72-1.08) | 0.91 (0.74-1.12) | 0.94 (0.76-1.15) | |  |
| **Deprivation, current (per 1-SD)** | **0.80 (0.68-0.93)** | **0.84 (0.72-0.98)** | 0.95 (0.80-1.12) | |  |
| Deprivation, early life (per 1-SD) | 0.87 (0.74-1.02) | 0.91 (0.77-1.08) | 0.94 (0.79-1.11) | |  |
| Unemployment, current | 1.02 (0.46-2.28) | 1.01 (0.45-2.25) | 1.23 (0.55-2.76) | |  |
| Unemployment, long term | 1.00 (0.54-1.87) | 1.06 (0.57-1.97) | 1.26 (0.67-2.36) | |  |
| No change in status since 1989 | 0.92 (0.56-1.51) | 0.90 (0.55-1.49) | 0.96 (0.58-1.59) | |  |
| Loss of status since 1989 | 0.87 (0.51-1.47) | 0.97 (0.57-1.65) | 1.07 (0.63-1.82) | |  |
| Age | 0.99 (0.97-1.02) | 1.00 (0.97-1.02) | 1.02 (0.99-1.05) | |  |
| **Male gender** | **1.77 (1.23-2.55)*** | 1.32 (0.88-1.97) | **1.67 (1.10-2.53)** | |  |
| *^a^ Adjusted for Age, sex, country, male*Russian interaction* | | | | | |
| *^b^ Adjusted for Age; sex; country; male*Russian interaction; diabetes; smoking; blood pressure; cholesterol; HDL; BMI; physical activity;*  *alcohol intake, frequency, binge pattern and problems.* | | | | | |
| *^c^ Adjusted for Age; sex; country; male*Russian interaction; diabetes; smoking; blood pressure; cholesterol; HDL; BMI; physical activity;*  *alcohol intake, frequency, binge pattern and problems; marital status; seeing relatives; seeing friends; friends*gender interaction; depression; material amenities; current unemployment.*  ** P value < 0.0036 (i.e. Bonferroni adjustment of 0.05/14).* | | | | | |
|  |  |  |  |  |  |
