# Supplementary material for: Psychosocial and socioeconomic determinants of cardiovascular mortality in Eastern Europe: A multicentre prospective cohort study
Source: PLoS Med. 2017 Dec 6;14(12):e1002459. doi: 10.1371/journal.pmed.1002459 (PMC5718419; doi:10.1371/journal.pmed.1002459)
Supplement: S8 Table — Data shows associations for 326 events, among those with no missing data on any covariate (N = 13,727). (DOCX) [file pmed.1002459.s009.docx]

| **S8 Table. Psychosocial factors and cardiovascular mortality: complete cases.**  Data shows associations for 326 events, among those with no missing data on any covariate (*N* = 13,727). | | | | | | | |  |
| --- | --- | --- | --- | --- | --- | --- | --- | --- |
|  |  |  |  |  |  |  |  |  |
|  |  |  |  |  |  |  |  |  |
|  | Hazard Ratios (95% confidence interval) | | |  | |  | |  |
|  | Model 1*^a^* | Model 2*^b^* | Model 3*^c^* | |  | |  | |
| *Psychosocial factors* |  |  |  | |  | |  | |
| Marital Status |  |  |  | |  | |  | |
| Married/cohabiting | 1 | 1 | 1 | |  | |  | |
| Divorced/widowed | **1.90 (1.45-2.50)** | **1.62 (1.23-2.13)** | 1.32 (0.99-1.75) | |  | |  | |
| Single | **2.62 (1.63-4.20)** | **2.52 (1.56-4.08)** | **1.90 (1.16-3.11)** | |  | |  |  |
| Social Support |  |  |  | |  | |  | |
| Contacts relatives <once/month | **1.55 (1.21-1.97)** | **1.40 (1.10-1.79)** | **1.37 (1.07-1.77)** | |  | |  | |
| Contacts friends <once/month | **0.72 (0.54-0.96)** | **0.72 (0.54-0.96)** | **0.66 (0.49-0.88)** | |  | |  | |
| friends*female interaction | **2.33 (1.42-3.83)** | **2.32 (1.41-3.83)** | **2.28 (1.38-3.77)** | |  | |  | |
| Not a member of a club | **1.53 (1.09-2.16)** | 1.23 (0.87-1.74) | 1.14 (0.81-1.62) | |  | |  | |
| Depression case | **1.97 (1.53-2.53)** | **1.74 (1.35-2.25)** | **1.49 (1.14-1.94)** | |  | |  | |
| Low perceived control (per 1-SD) | **1.24 (1.11-1.39)** | **1.13 (1.01-1.26)** | 0.98 (0.87-1.11) | |  | |  | |
| *Socioeconomic factors* |  |  |  | |  | |  | |
| Education |  |  |  | |  | |  | |
| Tertiary | 1 | 1 | 1 | |  | |  | |
| Secondary | **1.50 (1.14-1.98)** | 1.15 (0.87-1.53) | 1.00 (0.74-1.33) | |  | |  | |
| Primary | **2.11 (1.43-3.13)** | 1.35 (0.91-2.03) | 1.00 (0.66-1.54) | |  | |  |  |
| Material possessions |  |  |  | |  | |  | |
| Amenities, current (per 1-SD) | **1.58 (1.40-1.79)** | **1.38 (1.22-1.57)** | **1.22 (1.07-1.40)** | |  | |  | |
| Amenities, early life (per 1-SD) | 0.97 (0.84-1.12) | 0.95 (0.82-1.10) | 0.90 (0.77-1.04) | |  | |  | |
| Deprivation, current (per 1-SD) | **1.19 (1.07-1.34)** | 1.09 (0.97-1.22) | 0.92 (0.82-1.05) | |  | |  | |
| Deprivation, early life (per 1-SD) | 1.08 (0.97-1.21) | 1.05 (0.94-1.18) | 1.01 (0.90-1.13) | |  | |  | |
| Unemployment, current | **3.14 (1.91-5.18)** | **2.35 (1.40-3.92)** | **1.81 (1.07-3.04)** | |  | |  | |
| Unemployment, long term | **1.78 (1.18-2.68)** | **1.53 (1.01-2.30)** | 1.03 (0.63-1.67) | |  | |  | |
| Improvement in status since 1989 | 1 | 1 | 1 | |  | |  | |
| No change in status since 1989 | 1.00 (0.75-1.34) | 0.89 (0.66-1.19) | 0.78 (0.58-1.05) | |  | |  | |
| Loss of status since 1989 | 1.21 (0.88-1.66) | 1.07 (0.78-1.47) | 0.83 (0.60-1.16) | |  | |  | |
| *^a^ Adjusted for Age, sex, country, male*Russian interaction* | | | |  | |  | |  |
| *^b^ Adjusted for Age; sex; country; male*Russian interaction; diabetes; smoking; blood pressure; cholesterol; HDL; BMI; physical activity;*  *alcohol intake, frequency, binge pattern and problems.*  *^c^ Adjusted for Age; sex; country; male*Russian interaction; diabetes; smoking; blood pressure; cholesterol; HDL; BMI; physical activity;*  *alcohol intake, frequency, binge pattern and problems; marital status; seeing relatives; seeing friends; friends*gender interaction;*  *depression; material amenities; current unemployment.* | | | | | | | |  |
