# Supplementary material for: Psychosocial and socioeconomic determinants of cardiovascular mortality in Eastern Europe: A multicentre prospective cohort study
Source: PLoS Med. 2017 Dec 6;14(12):e1002459. doi: 10.1371/journal.pmed.1002459 (PMC5718419; doi:10.1371/journal.pmed.1002459)
Supplement: S9 Table — Data shows associations for 458 events, among those with at least two years of follow-up (N = 20,560). (DOCX) [file pmed.1002459.s010.docx]

| **S9 Table. Psychosocial factors and cardiovascular mortality: excluding 2 years.**  Data shows associations for 458 events, among those with at least 2 years of follow-up (*N* = 20,560). | | | | |
| --- | --- | --- | --- | --- |
|  |  |  |  |  |
|  |  |  |  |  |
|  | Hazard Ratio (95% confidence interval) | | |  |
|  | Model 1*^a^* | Model 2*^b^* | Model 3*^c^* |  |
| *Psychosocial factors* |  |  |  |  |
| Marital Status: |  |  |  |  |
| Married/cohabiting | 1 | 1 | 1 |  |
| Divorced/widowed | **1.57 (1.24-1.97)** | **1.32 (1.04-1.67)** | 1.06 (0.83-1.35) |  |
| Single | **2.59 (1.77-3.81)** | **2.42 (1.64-3.57)** | **1.72 (1.15-2.58)** |  |
| Social Support |  |  |  |  |
| Contacts relatives <once/month | **1.42 (1.15-1.74)** | **1.29 (1.05-1.58)** | **1.24 (1.00-1.54)** |  |
| Contacts friends <once/month | **0.77 (0.61-0.98)** | **0.75 (0.59-0.95)** | **0.67 (0.53-0.86)** |  |
| friends*female interaction | **2.16 (1.43-3.26)** | **2.16 (1.43-3.27)** | **2.14 (1.42-3.24)** |  |
| Not a member of a club | **1.60 (1.20-2.13)** | 1.31 (0.98-1.76) | 1.23 (0.92-1.65) |  |
| Depression case | **1.84 (1.47-2.31)** | **1.65 (1.29-2.11)** | **1.43 (1.11-1.84)** |  |
| Low perceived control (per 1-SD) | **1.29 (1.17-1.41)** | **1.18 (1.07-1.29)** | 1.03 (0.93-1.14) |  |
| *Socioeconomic factors* |  |  |  |  |
| Education |  |  |  |  |
| Tertiary | 1 | 1 | 1 |  |
| Secondary | **1.89 (1.45-2.47)** | **1.52 (1.16-1.99)** | **1.27 (0.96-1.67)** |  |
| Primary | **2.78 (2.01-3.83)** | **1.85 (1.33-2.57)** | **1.29 (0.91-1.83)** |  |
| Material possessions |  |  |  |  |
| Amenities, current (per 1-SD) | **1.64 (1.48-1.82)** | **1.45 (1.30-1.61)** | **1.31 (1.17-1.46)** |  |
| Amenities, early life (per 1-SD) | 0.98 (0.86-1.11) | 0.97 (0.86-1.10) | 0.93 (0.82-1.06) |  |
| Deprivation, current (per 1-SD) | **1.27 (1.16-1.39)** | **1.18 (1.08-1.29)** | 1.02 (0.92-1.13) |  |
| Deprivation, early life (per 1-SD) | **1.11 (1.01-1.22)** | 1.09 (0.99-1.20) | 1.03 (0.94-1.14) |  |
| Unemployment, current | **3.21 (2.04-5.04)** | **2.55 (1.62-4.03)** | **1.91 (1.20-3.04)** |  |
| Unemployment, long term | **2.01 (1.42-2.83)** | **1.75 (1.24-2.47)** | 1.25 (0.84-1.86) |  |
| Improvement in status since 1989 | 1 | **1** | 1 |  |
| No change in status since 1989 | 1.22 (0.95-1.58) | **1.10 (0.85-1.42)** | 0.93 (0.71-1.20) |  |
| Loss of status since 1989 | **1.38 (1.04-1.83)** | **1.19 (0.89-1.58)** | 0.86 (0.64-1.16) |  |
| *^a^ Adjusted for Age, sex, country, male*Russian interaction* | | | |  |
| *^b^ Adjusted for Age; sex; country; male*Russian interaction; diabetes; smoking; blood pressure; cholesterol; HDL; BMI; physical activity;*  *alcohol intake, frequency, binge pattern and problems.*  *^c^ Adjusted for Age; sex; country; male*Russian interaction; diabetes; smoking; blood pressure; cholesterol; HDL; BMI; physical activity;*  *alcohol intake, frequency, binge pattern and problems; marital status; seeing relatives; seeing friends; friends*gender interaction;*  *depression; material amenities; current unemployment.* | | | | |
