# Supplementary material for: Psychosocial and socioeconomic determinants of cardiovascular mortality in Eastern Europe: A multicentre prospective cohort study
Source: PLoS Med. 2017 Dec 6;14(12):e1002459. doi: 10.1371/journal.pmed.1002459 (PMC5718419; doi:10.1371/journal.pmed.1002459)
Supplement: S10 Table — Data shows associations for 514 events, among all participants (N = 20,867), whereby follow-up time was censored at 8.0 years in all three countries. (DOCX) [file pmed.1002459.s011.docx]

| **S10 Table. Psychosocial factors and cardiovascular mortality: limiting follow-up to eight years.**  Data shows associations for 514 events, among all participants (*N* = 20,867), whereby follow-up time was censored at 8.0 years in all 3 countries. | | | | |
| --- | --- | --- | --- | --- |
|  |  |  |  |  |
|  |  |  |  |  |
|  | Hazard Ratio (95% confidence interval) | | |  |
|  | Model 1*^a^* | Model 2*^b^* | Model 3*^c^* |  |
| *Psychosocial factors* |  |  |  |  |
| Marital Status: |  |  |  |  |
| Married/cohabiting | 1 | 1 | 1 |  |
| Divorced/widowed | **1.81 (1.46-2.24)** | **1.53 (1.23-1.90)** | **1.25 (1.00-1.57)** |  |
| Single | **2.60 (1.81-3.72)** | **2.41 (1.67-3.47)** | **1.80 (1.23-2.63)** |  |
| Social Support |  |  |  |  |
| Contacts relatives <once/month | **1.47 (1.22-1.78)** | **1.35 (1.11-1.63)** | **1.29 (1.06-1.58)** |  |
| Contacts friends <once/month | 0.84 (0.67-1.04) | 0.83 (0.67-1.03) | **0.74 (0.59-0.93)** |  |
| friends*female interaction | **2.16 (1.43-3.26)** | **1.84 (1.25-2.27)** | **1.84 (1.25-2.73)** |  |
| Not a member of a club | **1.75 (1.30-2.35)** | 1.42 (1.06-1.92) | 1.33 (0.98-1.79) |  |
| Depression case | **1.76 (1.42-2.17)** | **1.55 (1.23-1.94)** | **1.31 (1.02-1.67)** |  |
| Low perceived control (per 1-SD) | **1.31 (1.20-1.43)** | **1.20 (1.10-1.31)** | 1.06 (0.96-1.17) |  |
| *Socioeconomic factors* |  |  |  |  |
| Education |  |  |  |  |
| Tertiary | 1 | 1 | 1 |  |
| Secondary | **1.61 (1.27-2.04)** | **1.27 (1.00-1.61)** | 1.06 (0.83-1.36) |  |
| Primary | **2.59 (1.95-3.46)** | **1.68 (1.25-2.26)** | 1.20 (0.88-1.64) |  |
| Material possessions |  |  |  |  |
| Amenities, current (per 1-SD) | **1.62 (1.47-1.79)** | **1.42 (1.29-1.57)** | **1.26 (1.13-1.40)** |  |
| Amenities, early life (per 1-SD) | 1.01 (0.90-1.13) | 1.00 (0.89-1.23) | 0.95 (0.85-1.07) |  |
| Deprivation, current (per 1-SD) | **1.22 (1.13-1.32)** | **1.23 (1.04-1.23)** | 0.98 (0.89-1.07) |  |
| Deprivation, early life (per 1-SD) | **1.13 (1.04-1.23)** | **1.11 (1.01-1.21)** | 1.05 (0.96-1.15) |  |
| Unemployment, current | **2.87 (1.88-4.38)** | **2.25 (1.47-3.45)** | **1.74 (1.12-2.68)** |  |
| Unemployment, long term | **1.73 (1.24-2.42)** | **1.50 (1.07-2.10)** | 1.09 (0.74-1.61) |  |
| Improvement in status since 1989 | 1 | **1** | 1 |  |
| No change in status since 1989 | 1.13 (0.88-1.44) | 1.10 (0.78-1.27) | 0.84 (0.66-1.07) |  |
| Loss of status since 1989 | **1.34 (1.03-1.74)** | 1.13 (0.87-1.47) | 0.82 (0.62-1.08) |  |
| *^a^ Adjusted for Age, sex, country, male*Russian interaction* | | | |  |
| *^b^ Adjusted for Age; sex; country; male*Russian interaction; diabetes; smoking; blood pressure; cholesterol; HDL; BMI; physical activity;*  *alcohol intake, frequency, binge pattern and problems.* | | | | |
| *^c^ Adjusted for Age; sex; country; male*Russian interaction; diabetes; smoking; blood pressure; cholesterol; HDL; BMI; physical activity;*  *alcohol intake, frequency, binge pattern and problems; marital status;* *seeing relatives; seeing friends; friends*gender interaction; depression; material amenities; current unemployment.* | | | | |
|  |  |  |  |  |
