# Supplementary material for: Psychosocial and socioeconomic determinants of cardiovascular mortality in Eastern Europe: A multicentre prospective cohort study
Source: PLoS Med. 2017 Dec 6;14(12):e1002459. doi: 10.1371/journal.pmed.1002459 (PMC5718419; doi:10.1371/journal.pmed.1002459)
Supplement: S11 Table — 1,572 events among 20,867 participants. (DOCX) [file pmed.1002459.s012.docx]

| **S11 Table. Psychosocial factors and all-cause mortality.**  1572 events among 20,867 participants. | | | | | |
| --- | --- | --- | --- | --- | --- |
|  |  |  |  |  |  |
|  | Hazard Ratios (95% confidence interval) | | |  |  |
|  | Model 1*^a^* | Model 2*^b^* | Model 3*^c^* |  |  |
| *Psychosocial factors* |  |  |  |  |  |
| Marital Status |  |  |  |  |  |
| Married/cohabiting | **1** | **1** | **1** |  |  |
| Divorced/widowed | **1.61 (1.42-1.82)** | **1.38 (1.21-1.56)** | **1.18 (1.03-1.34)** |  |  |
| Single | **2.24 (1.81-2.77)** | **2.02 (1.63-2.50)** | **1.59 (1.27-1.99)** |  |  |
| Social Support |  |  |  |  |  |
| Contacts relatives < 1/month | **1.31 (1.05-1.63)** | **1.25 (1.12-1.40)** | **1.19 (1.06-1.34)** |  |  |
| Contacts friends < 1/month | 0.98 (0.87-1.11) | 0.95 (0.84-1.08) | 0.88 (0.77-1.00) |  |  |
| friends*female interaction | 1.22 (0.99-1.49) | **1.31 (1.05-1.64)** | **1.32 (1.06-1.65)** |  |  |
| Not a member of a club | **1.53 (1.31-1.77)** | **1.32 (1.13-1.53)** | **1.23 (1.06-1.44)** |  |  |
| Depression case | **1.72 (1.53-1.94)** | **1.54 (1.35-1.75)** | **1.34 (1.17-1.53)** |  |  |
| Low perceived control (per 1-SD) | **1.27 (1.21-1.33)** | **1.17 (1.11-1.23)** | 1.04 (0.98-1.10) |  |  |
| *Socioeconomic factors* |  |  |  |  |  |
| Education |  |  |  |  |  |
| Tertiary | **1** | **1** | **1** |  |  |
| Secondary | **1.61 (1.40-1.85)** | **1.36 (1.18-1.56)** | **1.15 (1.00-1.33)** |  |  |
| Primary | **2.39 (2.01-2.84)** | **1.76 (1.48-2.09)** | **1.28 (1.07-1.55)** |  |  |
| Material possessions |  |  |  |  |  |
| Amenities, current (per 1-SD) | **1.48 (1.40-1.56)** | **1.33 (1.26-1.41)** | **1.19 (1.12-1.27)** |  |  |
| Amenities, early life (per 1-SD) | 0.98 (0.92-1.05) | 0.97 (0.91-1.04) | 0.93 (0.87-1.00) |  |  |
| Deprivation, current (per 1-SD) | **1.23 (1.17-1.29)** | **1.14 (1.09-1.20)** | 1.00 (0.95-1.06) |  |  |
| Deprivation, early life (per 1-SD) | **1.10 (1.05-1.16)** | **1.08 (1.02-1.13)** | 1.03 (0.98-1.08) |  |  |
| Unemployment, current | **2.59 (2.04-3.28)** | **2.12 (1.67-2.70)** | **1.72 (1.35-2.20)** |  |  |
| Unemployment, long term | **1.68 (1.39-2.03)** | **1.46 (1.20-1.76)** | 1.07 (0.86-1.33) |  |  |
| Improvement in status since 1989 | **1** | **1** | 1 |  |  |
| No change in status since 1989 | **1.30 (1.13-1.49)** | **1.19 (1.03-1.36)** | 1.03 (0.89-1.18) |  |  |
| Loss of status since 1989 | **1.67 (1.44-1.94)** | **1.45 (1.24-1.69)** | 1.09 (0.98-1.28) |  |  |
| *^a^ Adjusted for Age, sex, country, male*Russian interaction* | | | |  |  |
| *^b^ Adjusted for Age; sex; country; male*Russian interaction; diabetes; smoking; blood pressure; cholesterol; HDL; BMI; physical activity;*  *alcohol intake, frequency, binge pattern and problems.* | | | | | |
| *^c^ Adjusted for Age; sex; country; male*Russian interaction; diabetes; smoking; blood pressure; cholesterol; HDL; BMI; physical activity;*  *alcohol intake, frequency, binge pattern and problems; marital status; seeing relatives; seeing friends; friends*gender interaction; depression; material amenities; current unemployment.* | | | | | |
|  |  |  |  |  |  |
