# Supplementary material for: Psychosocial and socioeconomic determinants of cardiovascular mortality in Eastern Europe: A multicentre prospective cohort study
Source: PLoS Med. 2017 Dec 6;14(12):e1002459. doi: 10.1371/journal.pmed.1002459 (PMC5718419; doi:10.1371/journal.pmed.1002459)
Supplement: S12 Table — Hazard ratios for cardiovascular mortality (in rows) attenuate from left to right, following the sequential addition of psychosocial covariates (columns). 556 events among 20,867 participants. (DOCX) [file pmed.1002459.s013.docx]

| **S12 Table. Attenuation of Psychosocial factors.**  Hazard Ratios (HR) for cardiovascular mortality (in rows) attenuate from left to right, following the sequential addition of psychosocial covariates (columns).  556 events among 20,867 participants. | | | | | | | | | | | | | | |  |  |  |  |  |  |  |  |
| --- | --- | --- | --- | --- | --- | --- | --- | --- | --- | --- | --- | --- | --- | --- | --- | --- | --- | --- | --- | --- | --- | --- |
|  |  |  |  |  |  |  |  |  |  |  |  |  |  |  |  |  |  |  |  |  |  |  |
|  |  | Model 1*^a^* | Model 2*^b^* | Model 2*^b^* plus one Psychosocial factor | | | | Model 2*^b^*  plus 4 Psychosocial factors | Model 2*^b^* plus one socioeconomic factor | | | Model 2*^b^*  plus 2 socioeconomic factors | Model 3*^c^* | |  |  |  |  |  |  |  |  |
|  |  |  |  | *^b^*+Single | *^b^*+Relatives | *^b^*+Friends | *^b^*+Depression |  | *^b^*+Material Amenities | *^b^*+Unemployed | |  |  |  |  |  |  |  |  |  |  |  |
| Education  (Primary vs. tertiary) | HR | 2.65 | 1.75 | 1.73 | 1.72 | 1.74 | 1.66 | 1.60 | 1.30 | 1.56 | | 1.22 | 1.24 | |  |  |  |  |  |  |  |  |
|  | Attenu-ation | ref. | -43% |  |  |  |  |  |  |  | |  |  | |  |  |  |  |  |  |  |  |
|  |  |  | ref. | -2% | -3% | -1% | -9% | -16% | -53% | -21% | | -64% | -62% | |  |  |  |  |  |  |  |  |
| Unemploy-ment | HR | 2.96 | 2.35 | 2.17 | 2.36 | 2.32 | 2.20 | 2.06 | 1.93 | n.a. | | 1.93 | 1.80 | |  |  |  |  |  |  |  |  |
|  | Attenu-ation | ref. | -21% |  |  |  |  |  |  |  | |  |  | |  |  |  |  |  |  |  |  |
|  |  |  | ref. | -9% | 0% | -2% | -8% | -15% | -23% |  | | -23% | -31% | |  |  |  |  |  |  |  |  |
| Material amenities (1-SD) | HR | 1.62 | 1.42 | 1.36 | 1.41 | 1.43 | 1.38 | 1.32 | n.a. | 1.36 | | 1.36 | 1.26 | |  |  |  |  |  |  |  |  |
|  | Attenu-ation | ref. | -27% |  |  |  |  |  |  |  | |  |  | |  |  |  |  |  |  |  |  |
|  |  |  | ref. | -12% | -2% | 2% | -8% | -21% |  | -12% | | -12% | -34% | |  |  |  |  |  |  |  |  |
| Depression case | HR | 1.82 | 1.62 | 1.56 | 1.58 | 1.63 | n.a. | 1.52 | 1.46 | 1.53 | | 1.41 | 1.38 | |  |  |  |  |  |  |  |  |
|  | Attenu-ation | ref. | -19% |  |  |  |  |  |  |  | |  |  | |  |  |  |  |  |  |  |  |
|  |  |  | ref. | -8% | -5% | 1% |  | -13% | -22% | -12% | | -29% | -33% | |  |  |  |  |  |  |  |  |
| Relatives  (less than monthly contact) | HR | 1.49 | 1.36 | 1.32 | n.a. | 1.40 | 1.31 | 1.32 | 1.30 | 1.37 | | 1.31 | 1.31 | |  |  |  |  |  |  |  |  |
|  | Attenu-ation | ref. | -23% |  |  |  |  |  |  |  | |  |  | |  |  |  |  |  |  |  |  |
|  |  |  | ref. | -10% |  | 9% | -12% | -10% | -15% | 2% | | -12% | -12% | |  |  |  |  |  |  |  |  |
| Single  (vs. married) | HR | 2.44 | 2.28 | n.a. | 2.19 | 2.27 | 2.20 | 2.12 | 1.74 | 1.56 | | 1.75 | 1.68 | |  |  |  |  |  |  |  |  |
|  | Attenu-ation | ref. | -8% |  |  |  |  |  |  |  | |  |  | |  |  |  |  |  |  |  |  |
|  |  |  | ref. |  | -5% | -1% | -4% | -9% | -33% | -46% | | -32% | -37% | |  |  |  |  |  |  |  |  |
| Friends (female participants only) | HR | 1.83 | 1.80 | 1.81 | 1.83 | n.a. | 1.77 | 1.81 | 1.81 | 1.79 | | 1.80 | 1.81 | |  |  |  |  |  |  |  |  |
|  | Attenu-ation | ref. | -3% |  |  |  |  |  |  |  | |  |  | |  |  |  |  |  |  |  |  |
|  |  |  | ref. | 1% | 3% |  | -3% | 1% | 1% | -1% | | 0% | 1% | |  |  |  |  |  |  |  |  |
| *^a^ Adjusted for Age, sex, country, male*Russian interaction* | | | | |  |  |  |  |  |  | |  |  | |  |  |  |  |  |  |  |  |
| *^b^ Adjusted for Age; sex; country; male*Russian interaction; diabetes; smoking; blood pressure; cholesterol; HDL; BMI; physical activity;*  *alcohol intake, frequency, binge pattern and problems.* | | | | | | | | | | | | |  | |  |  |  |  |  |  |  |  |
| *^c^ Adjusted for Age; sex; country; male*Russian interaction; diabetes; smoking; blood pressure; cholesterol; HDL; BMI; physical activity;*  *alcohol intake, frequency, binge pattern and problems; marital status; seeing relatives; seeing friends; friends*gender interaction; depression; material amenities; current unemployment.* | | | | | | | | | | |  | |  |  | | | |  |  |  |  |  |
|  |  |  |  |  |  |  |  |  |  |  |  | |  |  | | | |  |  |  |  |  |
