# Supplementary material for: Psychosocial and socioeconomic determinants of cardiovascular mortality in Eastern Europe: A multicentre prospective cohort study
Source: PLoS Med. 2017 Dec 6;14(12):e1002459. doi: 10.1371/journal.pmed.1002459 (PMC5718419; doi:10.1371/journal.pmed.1002459)
Supplement: S13 Table — Hazard ratios for cardiovascular mortality from being in Russia (versus being in Central Europe, shown in rows) attenuates from left to right, following the sequential addition of psychosocial covariates (columns). 556 events among 20,867 participants. Results are stratified for men (380 events) and women (176 events). (DOCX) [file pmed.1002459.s014.docx]

|  |  |  |  |  |  |  |  |  |
| --- | --- | --- | --- | --- | --- | --- | --- | --- |

| **S13 Table. Attenuation of country-dummies.**  Hazard Ratios (HR) for cardiovascular mortality, from being in Russia (versus. being in Central Europe, shown in rows), attenuates from left to right, following the sequential addition of psychosocial covariates (columns). 556 events among 20,867 participants. Results are stratified for men (380 events) and women (176 events). | | | | | | | | | | | | | | | | | | | | | | | | | | | | | | | | |  |  |  |  |
| --- | --- | --- | --- | --- | --- | --- | --- | --- | --- | --- | --- | --- | --- | --- | --- | --- | --- | --- | --- | --- | --- | --- | --- | --- | --- | --- | --- | --- | --- | --- | --- | --- | --- | --- | --- | --- |
|  |  |  |  |  |  |  |  |  |  |  |  |  |  |  |  |  |  |  |  |  |  |  |  |  |  |  |  |  |  |  |  |  |  |  |  |  |
|  |  |  |  | |  | |  |  | |  | |  | |  |  | |  | |  | | |  | | | | |  | | | | | | |  |  |  |
| Stratified sample |  | | Model 1*^a^* | Model 2*^b^* | | Model 2*^b^* plus one Psychosocial factor | | | | | | | | Model 2*^b^*  plus 4 Psychosocial factors | | Model 2*^b^* plus one socioeconomic factor | | | | Model 2*^b^*  plus 2 socioeconomic factors | | | | | | Model 3*^c^* | | | | | | |  |  |  |  |
|  |  | |  |  |  | ^b^+Single | | | ^b^+Relatives | | ^b^+Friends | | ^b^+Depression |  |  | ^b^+Amenities | | ^b^+Unemployed | |  |  |  |  |  |  |  |  |  |  |  |  |  |  |  |  |  |
| Male sample | HR | | 2.86 | 2.78 | | 2.84 | | | 2.74 | | 2.84 | | 2.76 | 2.89 | | 2.49 | | 2.95 | | 2.65 | | | | | | 2.77 | | | | | | |  |  |  |  |
|  | Attenuation | | ref. | -3% | | -1% | | | -4% | | -1% | | -3% | +1% | | -13% | | +3% | | -7% | | | | | | -3% | | | | | | |  |  |  |  |
| Female sample | HR | | 1.59 | 2.15 | | 2.07 | | | 2.07 | | 1.96 | | 2.04 | 1.79 | | 1.73 | | 2.22 | | 1.82 | | | | | | 1.64 | | | | | | |  |  |  |  |
|  | Attenuation | | ref. | +65% | | +57% | | | +57% | | +45% | | +54% | +26% | | +18% | | +72% | | +29% | | | | | | +7% | | | | | | |  |  |  |  |
| *^a^ Adjusted for Age, country.* | | | | | | | | | | | | | | | | | | | | | | | | |  | | | |  | |  | |  | | |  |
| *^b^ Adjusted for Age; country; diabetes; smoking; blood pressure; cholesterol; HDL; BMI; physical activity;*  *alcohol intake, frequency, binge pattern and problems.* | | | | | | | | | | | | | | | | | | | | | | | | |  | | | |  | |  | |  | | |  |
| *^c^ Adjusted for Age; country; diabetes; smoking; blood pressure; cholesterol; HDL; BMI; physical activity;*  *alcohol intake, frequency, binge pattern and problems; marital status; seeing relatives; seeing friends; friends*gender interaction; depression; material amenities; current unemployment.* | | | | | | | | | | | | | | | | | | | | |  | |  |  |  | | |  | |  | |  | | |  | |
|  |  |  |  |  |  |  |  |  |  |  |  |  |  |  |  |  |  |  |  |  |  | |  |  |  | | |  | |  | |  | | |  | |
